# The effect of 5‐HT_1A_ receptor agonists on the entopeduncular nucleus is modified in 6‐hydroxydopamine‐lesioned rats

**DOI:** 10.1111/bph.15437

**Published:** 2021-05-06

**Authors:** Sergio Vegas‐Suárez, Asier Aristieta, Catalina Requejo, Harkaitz Bengoetxea, José Vicente Lafuente, Cristina Miguelez, Luisa Ugedo

**Affiliations:** ^1^ Department of Pharmacology, Faculty of Medicine and Nursing University of the Basque Country (UPV/EHU) Leioa Spain; ^2^ Autonomic and Movement Disorders Unit, Neurodegenerative Diseases Biocruces Health Research Institute Barakaldo Spain; ^3^ Department of Biological Sciences Carnegie Mellon University Pittsburgh PA USA; ^4^ Center for the Neural Basis of Cognition Carnegie Mellon University Pittsburgh PA USA; ^5^ LaNCE, Department of Neuroscience University of the Basque Country (UPV/EHU) Leioa Spain

**Keywords:** 8‐OH‐DPAT, basal ganglia, buspirone, dyskinesia, electrophysiology, Parkinson's disease, 5‐hyroxytryptamine, serotonin

## Abstract

**Background and Purpose:**

l‐DOPA prolonged treatment leads to disabling motor complications as dyskinesia that could be decreased by drugs acting on 5‐HT_1A_ receptors. Since the internal segment of the *globus pallidus*, homologous to the entopeduncular nucleus in rodents, seems to be involved in the etiopathology of l‐DOPA‐induced dyskinesia, we investigated whether the entopeduncular nucleus is modulated by the 5‐HT_1A_ receptor partial and full agonists, buspirone, and 8‐hydroxy‐2‐(di‐n‐propylamino)‐tetralin (8‐OH‐DPAT) in control and 6‐hydroxydopamine (6‐OHDA)‐lesioned rats with or without long‐term l‐DOPA treatment.

**Experimental Approach:**

Extracellular single‐unit electrocorticogram and local field potential recordings under anaesthesia, immunostaining assays and optogenetic manipulation coupled to electrophysiological recordings were performed.

**Key Results:**

Systemic buspirone reduced the entopeduncular nucleus firing rate in the sham animals and burst activity in the 6‐OHDA‐lesioned rats (with or without l‐DOPA treatment), while local administration reduced entopeduncular nucleus activity in all the groups, regardless of DA integrity. Systemic 8‐OH‐DPAT also induced inhibitory effects only in the sham animals. Effects triggered by buspirone and 8‐OH‐DPAT were reversed by the 5‐HT_1A_ receptor antagonist, WAY‐100635. Neither buspirone nor 8‐OH‐DPAT modified the low‐frequency oscillatory activity in the entopeduncular nucleus or its synchronization with the motor cortex. Buspirone did not alter the response induced by subthalamic nucleus opto‐stimulation in the entopeduncular nucleus.

**Conclusion and Implications:**

Systemic 5‐HT_1A_ receptor activation elicits different effects on the electrophysiological properties of the entopeduncular nucleus depending on the integrity of the nigrostriatal pathway and it does not alter the relationship between subthalamic nucleus and entopeduncular nucleus neuron activity.

**Abbreviations**
6‐OHDA: 6‐hydroxydopamine
8‐OH‐DPAT: 8‐hydroxy‐2‐(di‐n‐propylamino)‐tetralin
DRD: dorsal raphe dorsal part; DRL; dorsal raphe lateral part
DRV: dorsal raphe ventral part
DRN: dorsal raphe nucleus
IOD: integrated optical densitometry
l‐DOPA: l‐3,4‐dihydroxyphenylalanine or levodopa
SERT: 5‐HT transporter

**What is already known**

- 5‐HT_1A_ receptor agonists are efficacious anti‐dyskinetic drugs.
- The entopeduncular nucleus suffers profound modifications in 6‐OHDA lesioned animals.

**What this study adds**

- Low doses of buspirone attenuates excessive burst activity in 6‐OHDA lesioned animals.

**What is the clinical significance**

- Anti‐dyskinetic properties of buspirone may partially rely on its effect on the entopeduncular nucleus.

## 1 INTRODUCTION

Parkinson's disease is characterized by the selective degeneration of the nigrostriatal pathway and a concomitant reduction in the striatal concentration of dopamine. The standard treatment for this loss of dopamine is the administration of the dopamine precursor, l‐3,4‐dihydroxyphenylalanine (l‐DOPA). However, prolonged treatment with l‐DOPA leads to disabling motor complications such as dyskinesia. When the l‐DOPA treatment losses efficacy or caused severe motor side effects, many patients move to deep brain stimulation treatment. Stimulation of the subthalamic nucleus or the internal part of the globus pallidus, which is homologous to the entopeduncular nucleus in rodents, reduces motor symptoms of Parkinson's disease and dyskinesia induced by prolonged administration of l‐DOPA (Olanow et al., 2003; Ramirez‐Zamora & Ostrem, 2018). The subthalamic nucleus and the internal part of the globus pallidus/entopeduncular nucleus are part of the basal ganglia nuclei. The subthalamic nucleus is the main excitatory source while the internal part of the globus pallidus/entopeduncular nucleus together with the substantia nigra reticulata constitutes the output nuclei, which receive a direct input from the subthalamic nucleus (Dudman & Gerfen, 2015). In Parkinson's disease patients and experimental models of Parkinson's disease, the subthalamic nucleus becomes hyperactive (Aristieta et al., 2012; Breit et al., 2007; Hassin‐Baer et al., 2011; Magill et al., 2001) and exaggerates the excitatory glutamatergic input to the internal part of the globus pallidus/entopeduncular nucleus, which also shows hyperactivity (Aristieta et al., 2019; Darbin et al., 2016; Jin et al., 2016). In addition, dopamine loss enhances oscillatory activity and synchronization within and between the basal ganglia nuclei, including the subthalamic nucleus and internal part of the globus pallidus and the cerebral cortex in distinct frequency bands. These alterations are related to motor manifestations in patients with Parkinson's disease (Alonso‐Frech et al., 2006). Overall these observations indicate that pharmacological modulation of the electrophysiological properties of the subthalamic nucleus and the internal part of the globus pallidus/entopeduncular nucleus could be a good alternative target for ameliorating Parkinson's disease‐related symptoms.

Among the limited number of pharmacological options for treatment of Parkinson's disease and dyskinesia, drugs acting on the 5‐hydroxytryptamine (5‐HT; serotonin) system are promising candidates for treating Parkinson's disease‐related symptoms (Miguelez et al., 2014). Although some findings have indicated that 5‐HT_1A_ receptor agonists may reduce anti‐parkinsonian l‐DOPA efficacy (Dupre et al., 2007; Iravani et al., 2006), buspirone, which is a partial agonist of the 5‐HT_1A_ receptor, appears to be a potential therapeutic agent (Loane & Politis, 2012) according to preclinical and clinical research of its anti‐parkinsonian and anti‐dyskinetic properties (Aristieta et al., 2012; Dekundy et al., 2007; Eskow et al., 2007; Politis et al., 2014). Furthermore, the efficacy of buspirone is currently being tested in four placebo‐controlled randomized double‐blind clinical trials, one phase III (NCT02617017), two phase II with co‐administration of the 5‐HT_1B/1D_ agonist zolmitriptan (NCT03956979 and NCT04377945) and one phase I as co‐therapy with amantadine (NCT02589340).

We have previously shown that buspirone has various effects on the subthalamic nucleus and substantia nigra reticulata, depending on the integrity of the nigrostriatal pathway (Sagarduy et al., 2016; Vegas‐Suárez et al., 2020). In addition, the activities of entopeduncular nucleus and subthalamic nucleus neurons were correlated in 6‐hydroxydopamine (6‐OHDA)‐lesioned rats treated with l‐DOPA (Aristieta et al., 2019). Continuing with this research and to characterize a good target for new pharmacological treatments for Parkinson's disease, we aimed to determine the effect of buspirone on entopeduncular nucleus neuron activity, oscillation and synchronization in control and 6‐OHDA‐lesioned rats with and without prolonged treatment with l‐DOPA and before and during subthalamic nucleus opto‐stimulation.

## 2 METHODS

### 2.1 Animals

A total of 87 (11 rats were excluded according to the results obtained in the cylinder test and the verification of the viral transfection within the subthalamic nucleus or because in the electrophysiological assay it was not possible to record the neuron after intravenous drug administration) 8‐week male Sprague–Dawley rats (RRID:MGI:5651135, SGIker facilities, UPV/EHU) weighing 150–175 g were housed in groups of at least four animals under standard laboratory (22 ± 1°C, 55 ± 5% relative humidity and a 12:12 h light/dark cycle) with *ad libitum* access to food and water. Animal studies are reported in compliance with the ARRIVE guidelines (Percie du Sert et al., 2020) and with the recommendations made by the *British Journal of Pharmacology* (Lilley et al., 2020), and they were approved by the Local Ethical Committee of the UPV/EHU (Protocol number: CEEA/M20/2016/176) following the European (2010/63/UE) and Spanish (RD 53/2013) regulations for the care and use of laboratory animals. We are aware of the importance of gender inclusion in preclinical research studies. However, we decided to use only male rats since 6‐OHDA‐lesioned rats show some differences between males and females (Sagarduy et al., 2016). Every effort was made to minimize animal suffering and to use the minimum number of animals per group and experiment.

### 2.2 Experimental design

**FIGURE 1:**
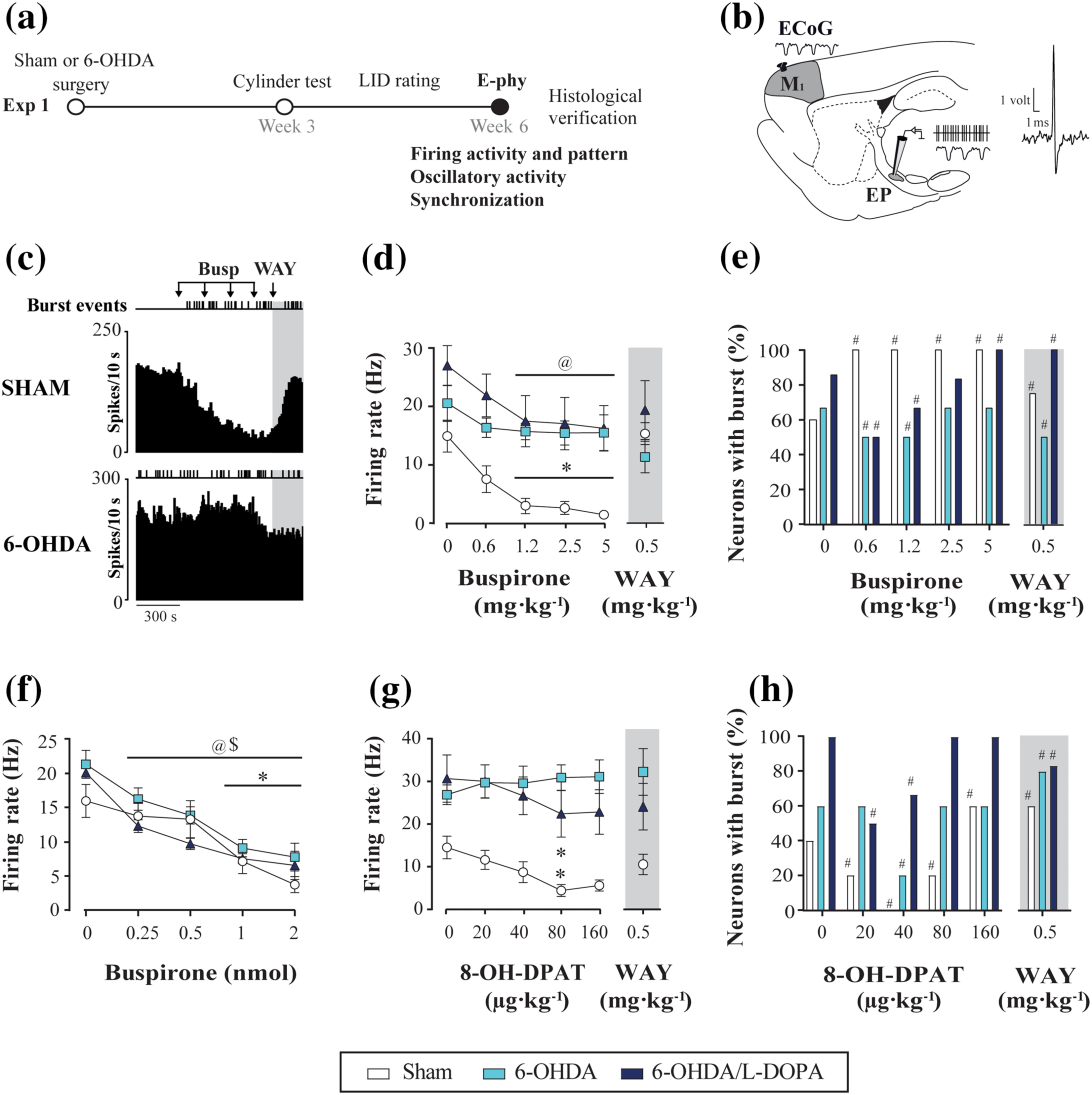
Effect of 5‐HT_1A_ receptor agonists on entopeduncular neuronal activity in sham and 6‐OHDAlesioned rats. (a) Experimental design of experiment 1. The rats received 6‐OHDA or vehicle injection into the medial forebrain bundle and the accuracy of the lesion was screened using the cylinder test 3 weeks later. Six weeks after the 6‐OHDA lesion, entopeduncular nucleus (EP) single‐unit extracellular electrophysiology (E‐phy) was performed. (b) Schematic parasagittal section of a rat brain, showing the motor cortex and a glass electrode placed in the EP for recording single‐unit extracellular activity and local field potential (LFP). On the right, an example of an EP neuron action potential. (c) Examples of the effects of buspirone systemic administration (0.6125–5 mg·kg^−1^, i.v.) on EP neuronal activity in sham and 6‐OHDA‐lesioned rats. (d–e) Corresponding dose‐effect curves of systemic buspirone (d) and the number of neurons showing burst activity (e) in sham (*n* = 5), 6‐OHDA (*n* = 6), and 6‐OHDA/l‐DOPA groups (*n* = 6). Note that the administration of WAY‐100635 reversed the inhibitory effect (grey shadow). (f) Local administration of buspirone (0.25–2 nmol) induced a dose‐dependent inhibitory effect in sham (*n* = 5), 6‐OHDA (*n* = 5), and 6‐OHDA/l‐DOPA (*n* = 5). (g, h) Graphics representing the firing rate (g) and the number of neurons exhibiting burst activity (h) after increasing doses of 8‐OH‐DPAT (20–160 μg·kg^−1^, i.v.) in sham (*n* = 5), 6‐OHDA (*n* = 5) and 6‐OHDA/l‐DOPA (*n* = 6). Data are expressed as mean ± S.E.M. *P < .05 (sham), ^@^ *P* < .05 (6‐OHDA/l‐DOPA), and ^$^ *P* < .05 (6‐OHDA) versus respective basal values (RM two‐way ANOVA followed by Bonferroni's post hoc test) and ^#^ *P* < .05 versus respective baseline (Fisher's exact test for firing pattern)

Rats were randomly divided into three different experimental groups:‐ (1) sham, (2) lesion with 6‐OHDA and (3) lesion with 6‐OHDA and chronically treated with l‐DOPA (6‐OHDA/l‐DOPA), in order to be able to assess whether possible observed changes on drug response were due to the effect of the 6‐OHDA lesion or to the effect of prolonged l‐DOPA treatment. For experiments 1 and 2 (electrophysiological and immunohistochemical assays), the rats received 6‐OHDA or vehicle injection into the medial forebrain bundle and the accuracy of the lesion was screened using the cylinder test 3 weeks later (Figures 1a and 3a). Only animals showing a reduction of striatal integrated optical densitometry (IOD) of tyrosine hydroxylase (TH IOD; >80%) were included in the 6‐OHDA group. After the cylinder test, a group of 6‐OHDA‐lesioned rats received l‐DOPA treatment for 3 weeks and abnormal involuntary movements were evaluated at the beginning and the end of the treatment. Electrophysiological recordings and/or perfusion were performed 6 weeks after the surgery (sham or 6‐OHDA groups) and 24 h after the last dose of l‐DOPA (6‐OHDA/l‐DOPA group). In experiment 3 (Figure 4a), optogenetic manipulation of the subthalamic nucleus was performed 4 weeks after the viral injection. Later, the viral transfection in the subthalamic nucleus was histologically confirmed. All experiments were analysed under blinded conditions according to *British Journal of Pharmacology* guidelines (Kilkenny et al., 2010).

### 2.3 Stereotaxic surgery

#### 2.3.1 6‐OHDA lesion

6‐OHDA lesions were carried out as described in detail in one of our well‐established protocols (Aristieta et al., 2019). Thirty minutes before surgery, the rats were pretreated with desipramine (25 mg·kg^−1^, i.p.) and pargyline (50 mg·kg^−1^, i.p.) to prevent damage to the noradrenergic system or degradation of the neurotoxin, respectively. Next, the rats were deeply anaesthetized using isoflurane in medical oxygen grade 1l/min 99.5% (4% for induction and 1.5–2.0% for maintenance) and placed in a stereotaxic frame (David Kopf® Instruments) and were allowed to breath spontaneously. The surgical procedure was performed with sterilized material and povidone‐iodine was applied prior and after the stereotaxic injection for maintaining aseptic conditions. 6‐OHDA (3.5 μg·μl^−1^, in 0.02% ascorbic acid, max volume 4.5 μl) or vehicle was injected using a 10‐μl Hamilton syringe at a rate of 1 μl·min^−1^ in the co‐ordinates of the right medial forebrain bundle (Paxinos & Watson, 1997): 2.5 μl at the anteroposterior (AP) (−4.4 mm), mediolateral (ML) (+1.2 mm) and dorsoventral (DV) (−7.8 mm) relative to bregma with a toothbar set at −2.4 and 2 μl at AP (−4.0 mm), ML (+0.8 mm) and DV (−8 mm) with a toothbar at +3.4. After the surgery, animals were allowed to fully recover in individual cages. This animal model has been widely used and replicates pathophysiological aspects of Parkinson's disease in humans (Przedbroski et al., 1995).

#### 2.3.2 Viral vector injection

The rats were deeply anaesthetized with isoflurane and placed in the stereotaxic frame as above. The viral vector AAV5‐hSyn‐hChR2‐(H134R)‐EYFP (UNC Vector Core, USA) was delivered with a calibrated thick‐walled micropipette (7087‐07, BLAUBRAND®, intraMark, Germany) previously broken to a tip diameter of approximately 40 μm. Pulses (50–150 ms) were applied using Picospritzer™ II (General Valve Corporation, Fairfield, NJ, USA). A total volume of 500 nl of the viral vector was delivered to the subthalamic nucleus at the following coordinates (relative to bregma and dura matter): 250 nl at AP (−3.6 mm), ML (−2.5 mm) and DV (−7.5 mm) and 250 nl at AP (−3.9 mm), ML (−2.5 mm) and DV (−7.5 mm) (Paxinos & Watson, 1997). After the surgery, animals were allowed to fully recover in individual cages.

### 2.4 Behavioural tests

#### 2.4.1 Cylinder test

Three weeks after the 6‐OHDA lesion, when the animals had totally recovered, forelimb asymmetry was evaluated using the drug‐free test cylinder test as previously described (Miguelez et al., 2011). Each rat was left in the cylinder until reaching a total of 20 touches with any of both forelimbs on the walls or a maximum of 5‐min exploration time. The percentage of contralateral forelimb use with respect to the total number of contacts was calculated. Only animals with an ipsilateral contact percentage of ≥70% were enrolled in this study (Figure S1A).

#### 2.4.2 Long‐term l‐DOPA treatment and abnormal involuntary movement rating

6‐OHDA rats received daily treatment with l‐DOPA (6 mg·kg^−1^, i.p.) in combination with peripheral decarboxylase inhibitor benserazide (12 mg·kg^−1^, i.p.) over 3 weeks. At the beginning and end of the treatment, abnormal involuntary movements were scored (Figure S1B,C). Each rat was observed for one full minute every 20th min during the 200‐min testing period. The severity of each of the three subtypes of dyskinetic movements (axial, limb and orolingual) was rated from 0 to 4 based on the amount of time (0, not present to 4, continuous). In addition, the amplitude of each dyskinetic movement was rated on a scale from 0 to 4, separately from the locomotive movements (Cenci & Lundblad, 2007). All 6‐OHDA/l‐DOPA rats included in this study developed severe abnormal involuntary movements.

### 2.5 Electrophysiological procedures

#### 2.5.1 Single‐unit extracellular recordings of entopeduncular neurons

Electrophysiological recordings of entopeduncular nucleus neurons (Figure 1b) were performed 3 weeks after the surgery and 24 h after the last l‐DOPA injection in the 6‐OHDA/l‐DOPA group (Aristieta et al., 2019). The rats received one doses of urethane anaesthesia (1.2 g·kg^−1^, i.p.) and the right jugular vein was cannulated for systemic drug administration. Next, each rat was placed in a stereotaxic frame with its head secured in the horizontal plane. The skull was exposed and a bur‐hole was drilled over the co‐ordinates of the entopeduncular nucleus. The recording electrode, consisting of a single barrel glass micropipette (TW150F‐4, World Precision Instruments, UK) filled with a 2% solution of Pontamine Sky Blue in 0.5% sodium acetate, was lowered into the right entopeduncular nucleus (AP: −2.3 mm, ML: −2.5 mm, DV: −7.0 to 8.5 mm, relative to bregma and dura) (Paxinos & Watson, 1997) and neuronal spikes were digitized using the CED micro 1401 interface and Spike2 software (Cambridge Electronic Design). The firing rate was recorded for 300 s under basal conditions and 150 s after each systemic drug dose. These were injected (i.v.) at 1‐min intervals, in increasing (×2) doses, so that cumulative dose effect was analysed. Dose and interval for each drug was selected according to our previous results (Aristieta et al., 2014; Sagarduy et al., 2016; Vegas‐Suárez et al., 2020). To identify entopeduncular nucleus neurons, we took the following electrophysiological characteristics into account, biphasic action potential with a duration of 0.8–0.9 ms and firing rate between 10 and 20 Hz. For local administration, a calibrated pipette attached to a recording electrode was filled with 0.25‐M buspirone dissolved in Dulbecco's buffered solution, as previously described (Vegas‐Suárez et al., 2020). Local buspirone administration was performed by applying pressure pulses using a Picospritzer™ II. The injected volume was simultaneously monitored with the movement of the fluid in a calibrated pipette. Each pulse corresponded to the injection of 2 nl of solution and 0.25 nmol of buspirone. Every pipette was calibrated so that each pulse corresponded to the injection of 2 nl of solution (0.25 nM). Then, two pulses would be 0.5 nM, four pulses would be 1 nM and eight pulses would be 2 nM. Each drug application was performed when the effect of previous application on the neuronal firing rate was completely recovered.

#### 2.5.2 Oscillatory activity and synchronization

The electrocorticogram was simultaneously recorded in these urethane anesthetized rats via a 1‐mm‐diameter steel screw on the right frontal motor cortex (AP: +4.5 mm and ML: −2.5 mm, relative to bregma) (Paxinos & Watson, 1997). The signal was pre‐amplified (10×), amplified (200×) and bandpass filtered (0.1–1000 Hz) in an amplifier (Cibertec S.A., model amplifier 63AC). The local field potential was recorded with the same glass electrode that was used for the entopeduncular nucleus single‐unit extracellular recordings. The recording signal was pre‐amplified (10×) and amplified (10×) in a high‐input impedance amplifier (Cibertec S.A., model amplifier AE‐2) and bandpass filtered (0.1–5000 Hz). This signal was divided into two different signals in a second amplifier (63AC), namely, the single‐unit and the local field potential signals. In this second amplifier, the local field potential signal was amplified (10×) and bandpass filtered (0.1–100 Hz). The discriminated electrocorticogram and the local field potential activity were digitized and further analysed offline. At the end of the experiments, a 5‐μA cathodal current was passed through the recording electrode to create a discrete mark of the Pontamine Sky on the recording site, which would be used to verify the recording site. Experiments were terminated by transcardiac perfusion and brain extraction.

#### 2.5.3 Integrated *in vivo* optogenetic stimulation of the subthalamic nucleus coupled to the subthalamic and entopeduncular electrophysiological recordings

Four weeks after the viral transfection, subthalamic nucleus or entopeduncular nucleus recordings were performed as described during subthalamic nucleus optogenetic stimulation (Figure 4a). The optic bare fibre (200/230 μm core; 1.5‐m length, 94063, Plexon, Texas, USA) was inserted into a modified glass capillary and lowered into the transfected subthalamic nucleus at a 30° angle to the horizontal plane (relative to bregma and dura, AP: −8.23 mm, ML: −2.5 mm and DV: −8.66 mm) using a micromanipulator. The optic fibre was connected to a PlexBright 465‐nm blue LED for an optogenetic stimulation system (94002‐002, Plexon, Texas, USA). The stimulation was digitally controlled by Spike2 software and different protocols were designed (5.5 mV and 1 s continuous stimulation, 14 mW and 0.5 s continuous stimulation, 14 mW and 25 Hz trains of stimulation). Previously, the LED intensity was tested and calibrated using a digital power meter kit (PM100D, Thorlabs, New Jersey, USA). The intensity was set between 0.5 and 15 mW measured at the tip of the optic fibre (Figure 4c). Peristimulus time histograms were generated from at least 10 stimulation trials using 1‐ms bins to determine the type of response evoked by, as well as calculate the firing rate before (pre) and during (light‐on), the stimulation. The digital protocols were randomized and applied to each neuron and separated by 5 min. After the electrophysiological recordings, the verification of viral transfection in the subthalamic nucleus was performed using confocal microscopy (Zeiss LSM800) (Figure 4b). Only rats that expressed the appropriate viral transfection within the subthalamic nucleus and excitation in at least 75% of the stimulated cells were included in the study. Experiments were terminated by transcardiac perfusion and brain extraction.

#### 2.5.4 Analysis of electrophysiological data

Analysis of the electrophysiological parameters was performed offline using the Spike2 software (version 7). The firing rate and coefficient of variation were obtained by applying a script (“meanix.s2s”). The percentage of neurons demonstrating the burst firing pattern and burst‐related parameters (the number of bursts, mean duration of burst, spikes per burst, recurrence of burst and intraburst frequency) were calculated using the script (“surprise.s2s”), which was based on the Poisson Surprise method (minimum s value = 3 consecutive spikes), within 90 s (Vegas‐Suárez et al., 2020). As described in (Aristieta et al., 2019), local field potential and electrocorticogram signals were smoothed to 1 ms and the entopeduncular nucleus action potentials were converted to a series of events. Next, these events were transformed into a continuous waveform (1‐ms smoothing period) using a custom‐made script. Local field potential and electrocorticogram power spectra and the coherence between entopeduncular nucleus‐spikes and local field potentials, as well as entopeduncular nucleus‐spikes and electrocorticograms and local field potentials and electrocorticograms, were analysed using the fast Fourier transform (8192 blocks size) in the low‐frequency range (0–5 Hz) during 90 s applying a script (“coherac90.s2s”). The AUC of each curve was also calculated. When several neurons were recorded per animal, the values were averaged and represented as the only value.

### 2.6 Histological procedures and analyses

#### 2.6.1 Histochemical assays

The immuno‐related procedures used comply with the recommendations made by the *British Journal of Pharmacology* (Alexander et al., 2018). Animals were deeply anaesthetized (1.2 g·kg^−1^ urethane i.p.) and transcardially perfused with saline at 37°C followed by 4% ice‐cold paraformaldehyde and 0.2% picric acid prepared in a 0.1‐M phosphate saline buffer. The brains were removed and fixed overnight in paraformaldehyde. Twenty‐four hours later, the brains were transferred to a 25% sucrose solution until they sank and they were serially cut in coronal 40‐μm sections using a freezing microtome. Brain slices were kept in a cryoprotectant solution at −20°C until further processing. We performed tyrosine hydroxylase, 5‐HT transporter (SERT) and 5‐HT_1A_ receptor immunohistochemistry and neutral red staining.

Throsine‐immunochemistry was used to assess the degree of dopaminergic denervation in the striatum (Vegas‐Suárez et al., 2020). After endogenous peroxidase inactivation with 3% H_2_0_2_ and 10% methanol in potassium phosphate‐buffered saline (KPBS), the sections were pre‐incubated with 5% normal goat serum and incubated with primary antibody (rabbit anti‐TH, 1:1000, Merck Millipore, Spain) in KPBS/T containing 5% normal goat serum (NGS) overnight at 22°C. In the following days, the sections were rinsed and incubated for 2 h with the secondary antibody (biotinylated goat anti‐rabbit IgG, 1:200, Vector Laboratories, California, USA) in 2.5% NGS KPBS/T. Thereafter, the sections were incubated with an avidin‐biotin‐peroxidase complex (ABC‐kit, PK‐6100, Vector Laboratories, California, USA) and peroxidase activity was visualized using 0.05% 3,3‐diaminobenzidine (DAB) and 0.03% H_2_O_2_. The sections were mounted onto gelatine‐coated slides, dehydrated in an ascending series of ethanol and coverslipped in DPX mounting medium.

For another set of slices, we performed SERT and 5‐HT_1A_‐receptor immunohistochemistry following our established protocol (Vegas‐Suárez et al., 2020). We analysed basal ganglia areas such as the striatum (STR), nucleus accumbens, external globus pallidus, entopeduncular nucleus, subthalamic nucleus, substantia nigra and the dorsal, ventral or lateral parts (DRD, DRV and DRL) of the dorsal raphé nucleus (DRN). Briefly, after endogenous peroxidase inactivation, the sections containing the basal ganglia and DRN were blocked with BSA to prevent non‐specific antibody binding and incubated with the primary antibody (rabbit anti‐SERT, 1:2500, Immunostar, Hudson, WI, USA; or rabbit anti‐5‐HT_1A_, 1:200, Genetex, California, USA) for 48 h at 22°C. Next, the sections were rinsed and incubated with the secondary biotinylated antibody (donkey anti‐rabbit IgG, 1:400, Jackson Immunoresearch, Stratech Scientific; or goat anti‐rabbit IgG, BA‐1000, 1:200, Vector Laboratories, California, USA) for 2 h at 22°C. Sections were rinsed between antibody treatments to remove unbound and weakly bound antibodies. Later, the sections were incubated with an ABC‐kit and visualized using 0.022% DAB and 0.003% H_2_O_2_. Finally, the sections were mounted, dehydrated and coverslipped.

For the location of the recording site, coronal sections containing the entopeduncular nucleus were stained with 1% neutral red (Miguelez et al., 2011), washed, dehydrated and coverslipped. Only neurons recorded within the entopeduncular nucleus were included in the study.

#### 2.6.2 Integrated optical densitometry (IOD) of the basal ganglia and dorsal raphé nuclei

The IOD of tyrosine hydroxylase (TH) immunoreactivity in the striatum and SERT and 5‐HT_1A_ receptor immunoreactivity in the basal ganglia and DRN were measured as grey levels using NIH‐produced software: (ImageJ win64 Fiji (RRID:SCR_002285; https://imagej.net/Fiji)). Digital images were obtained using the 20× objective of an automatic panoramic digital slide scanner (Panoramic MIDI II, 3DHistech, Hungary) and CaseViewer 2.3 (64‐bit version) software (RRID:SCR_017654). The analysis was blinded. Two to four slices were used per nucleus and the mean IOD was determined by subtracting the background defined as a non‐immunoreactive zone. The results (IOD%) were expressed as the ratio of the optical intensities of the ipsilateral (right) lesioned hemisphere and the intact or contralateral (left) non‐lesioned or intact hemisphere. IOD of the nuclei in the contralateral hemisphere was similar among the groups, ruling out any compensatory effect due to the lesion or treatment (Table S1).

### 2.7 Data and statistical analysis

The data and statistics analysis comply with the recommendations and Declaration of Transparency and Scientific Rigour of *British Journal of Pharmacology* (Curtis et al., 2018). Experimental data were analysed using GraphPad Prism (RRID:SCR_002798, v. 5.01, GraphPad Software, Inc.). For the basal electrophysiological parameters, SERT and 5‐HT_1A_ receptor expressions, ANOVA was applied, except for the analysis of burst firing, which was conducted using Fisher's exact test. The effect of systemic or local drug administration on the electrophysiological parameters was analysed using repeated measures (RM) two‐way ANOVA (group × treatment). For the systemic administration of drugs, only one cell was recorded per animal. For local drug administration and optogenetic stimulation, several neurons were recorded from the same animals, the electrophysiological parameters values and data were averaged per animal, so that every animal had one value. For optogenetic experiments, the paired two‐tailed Student's *t*‐test was used to compare subthalamic nucleus activity before (pre) and during (light‐on) optogenetic stimulation. In addition, the effect of buspirone on entopeduncular nucleus neurons after and during subthalamic nucleus optogenetic stimulation was analysed using the repeated measures (RM) two‐way ANOVA. All ANOVAs were followed by Bonferroni's post hoc tests only if *F* was significant and there was no significant variance inhomogeneity. The threshold of statistical significance was set at *P* < .05, with corresponding 95% confidence intervals. All detailed statistical results are shown in Table S1. Animals were randomized and group size was ≥5 in all cases. Sometimes groups were unequal for loss of samples during the recording or the histological verification. Data were presented as the mean ± SEM for electrophysiological parameters or Box and Whiskers representing the median and min to max values for behavioural tests, IOD or optogenetics experiments. Normalization was used when analysing oscillatory activity and synchronization to avoid unwanted variation among the groups.

### 2.8 Materials

Desipramine hydrochloride, pargyline, l‐DOPA, benserazide hydrochloride and buspirone hydrochloride were obtained from Sigma‐Aldrich, while WAY‐100635 maleate and 8‐OH‐DPAT were obtained from Tocris‐Biogen. These drugs were prepared in 0.9% saline. Urethane and 6‐OHDA hydrobromide (Sigma‐Aldrich) were dissolved in Milli‐Q water and Milli‐Q water containing 0.02% ascorbic acid, respectively. All drug solutions were prepared on the day of the experiment.

### 2.9 Antibodies

As described above, the following primary antibodies were used: rabbit anti‐tyrosine hydroxylase (Cat#AB152, RRIB:AB_3902) for dopamine fibres, rabbit anti‐SERT (Cat#RA24330–100, RRID:AB_1622792) for the SERT and rabbit anti‐5‐HT_1A_ (Cat#GTX104703, RRID_AB1241_307) for the 5‐HT_1A_ receptor. Subsequently, the following biotinylated secondary polyclonal antibodies were used: donkey anti‐rabbit IgG, (Cat#711–005‐152, RRID:AB_2340) and goat anti‐rabbit IgG (Cat#BA‐1000, RRID:AB_2313606).

### 2.10 Nomenclature of targets and ligands

Key protein targets and ligands in this article are hyperlinked to corresponding entries in the IUPHAR/BPS Guide to PHARMACOLOGY http://www.guidetopharmacology.org and are permanently archived in the Concise Guide to PHARMACOLOGY 2019/20 (Alexander et al., 2019).

## 3 RESULTS

### 3.1 Entopeduncular nucleus neuron activity in sham and 6‐OHDA‐lesioned rats

For experiment 1, we recorded and analysed 90 GABAergic neurons from 53 anaesthetized rats (Figure 1a,b): 26 neurons from the sham group (*n* = 15), 27 cells from the 6‐OHDA group (*n* = 16) and 32 neurons from the 6‐OHDA/l‐DOPA group (*n* = 17). Post‐mortem histological verification and distinctive electrophysiological properties (described in the methods) indicated that all recorded cells were GABAergic neurons located within the entopeduncular nucleus.

**FIGURE 2:**
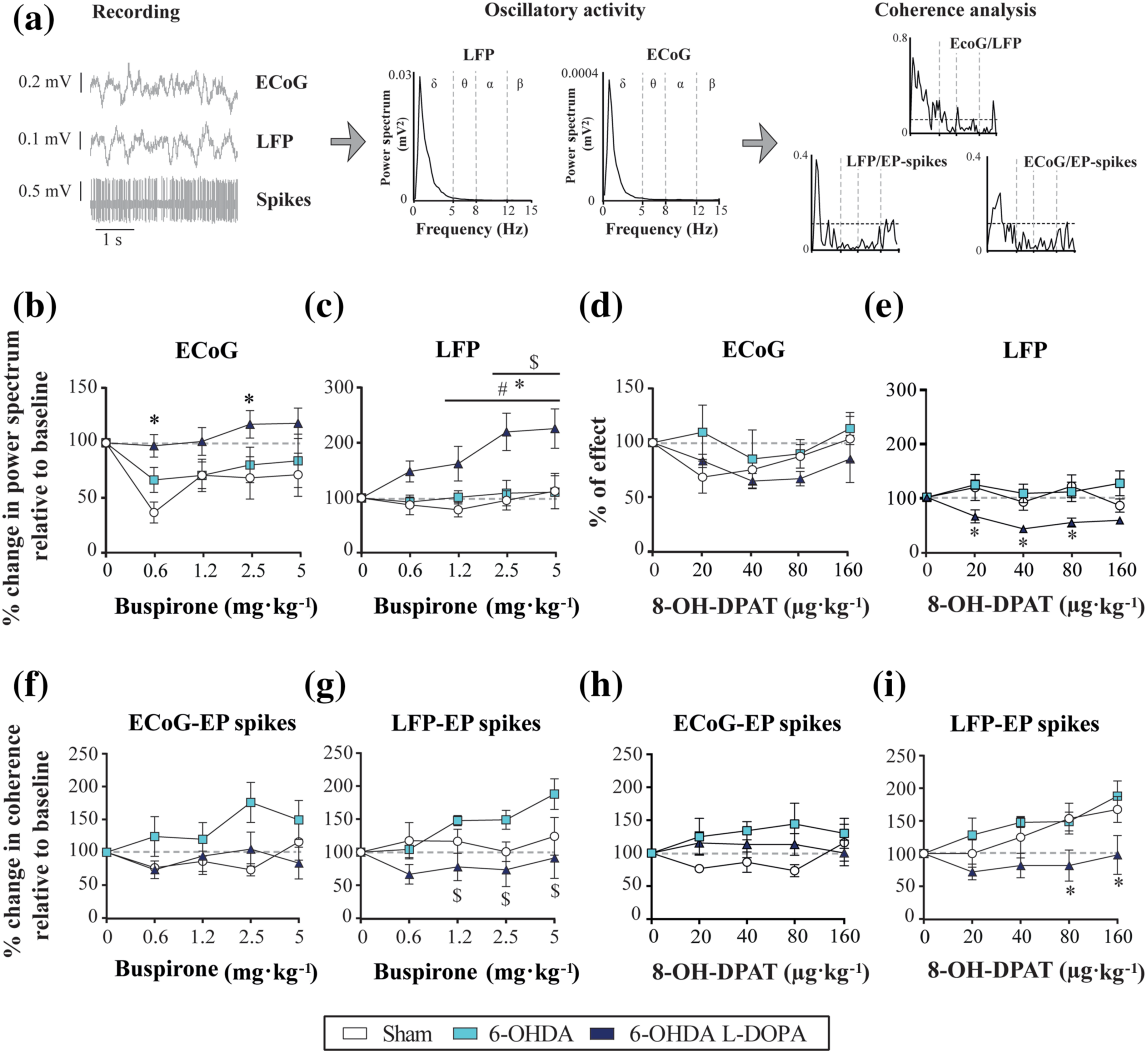
Effect of systemic administration of buspirone and 8‐OH‐DPAT on the oscillatory activity and synchronization of the entopeduncular nucleus and the motor cortex in the low‐frequency range (0–5 Hz). (a) Recording track containing entopeduncular nucleus (EP) spikes, EP‐local field potential (LFP) and electrocorticogram (ECoG) from a neuron in the sham group, and posterior analysis of the oscillatory activity and synchronization represented by the power spectra and coherence plots. (b, c) Effect of increasing doses of systemic buspirone (0.6125–5 mg·kg^−1^, i.v.) on the percentage of change in AUC of the power spectra relative to baseline values of the ECoG (b) and EP‐LFP (c) in the three experimental groups. (d, e) Same as b and c for 8‐OH‐DPAT (20–160 μg·kg^−1^, i.v.). (f, g) Effect of acute doses of buspirone on the percentage of change in the AUC of coherence relative to baseline values between ECoG and EP spikes (f) and EP‐LFP and EP spikes (g). (h, i) Same as f and g for 8‐OH‐DPAT (20–160 μg·kg^−1^, i.v.). Data are expressed as mean ± S.E.M. *P < .05 versus sham, ^$^ *P* < .05 versus 6‐OHDA and ^#^ *P* < .05 versus baseline (one‐way ANOVA or RM two‐way ANOVA followed by Bonferroni's post hoc test). Buspirone was tested in five (sham), six (6‐OHDA) and six (6‐OHDA/l‐DOPA) animals and 8‐OH‐DPAT in five (sham), five (6‐OHDA) and six (6‐OHDA/l‐DOPA) animals

Neurons in the 6‐OHDA/l‐DOPA group fired at higher frequencies and had a significant higher coefficient of variations, while the number of neurons exhibiting a burst firing pattern significantly increased in all the 6‐OHDA groups, regardless of l‐DOPA treatment (Table 1). Together with the entopeduncular nucleus single‐cell activity, we analysed the oscillatory activity and synchronization by simultaneously recording the motor cortex electrocorticogram, entopeduncular nucleus‐local field potential and single‐unit extracellular activity in the same groups described above. In agreement with our previous publication (Aristieta et al., 2019), power spectra analysis showed low oscillatory activity (0–5 Hz) in the electrocorticogram and local field potential with a peak near 1 Hz in the three experimental groups, under basal conditions (Figure 2a). As shown in Table 1, the AUC values of the electrocorticogram power spectrum were similar in the three experimental groups, whereas the local field potential AUC values were significantly higher in the 6‐OHDA group. The coherence analysis showed that synchronization between electrocorticogram and entopeduncular nucleus spikes, as well as the entopeduncular nucleus‐local field potential and entopeduncular nucleus spikes, was significantly higher in the 6‐OHDA/l‐DOPA group,while no difference was observed in the synchronization between electrocorticogram and entopeduncular nucleus‐local field potential among the studied groups.

**TABLE 1:** Entopeduncular nucleus (EP) electrophysiological properties

Parameters	Sham (*n* = 15)	6‐OHDA (*n* = 16)	6‐OHDA/l‐DOPA (*n* = 17)
Firing rate (Hz)	17.4 ± 1.7	24.6 ± 2.7	26.7 ± 2.7*
CV (%)	46.8 ± 6.0	55.0 ± 9.4	80.3 ± 10.8*
Neurons exhibiting burst firing pattern (%)	61.5	77.8^$^	87.5^$^
Number of bursts	46.8 ± 13.6	29.4 ± 10.9	46.9 ± 13.9
Duration of burst (ms)	0.6 ± 0.3	0.3 ± 0.1	0.3 ± 0.1
No. of spikes/burst	11.3 ± 2.5	14.1 ± 6.4	14.4 ± 2.2
Recurrence of burst (no. of burst/min)	33.3 ± 9.5	19.6 ± 7.4	24.6 ± 6.2
Intraburst frequency (spike/s)	71.5 ± 14.3	62.6 ± 8.9	66.2 ± 5.1
AUC of the power spectrum of the ECoG (AUC)	0.00052 ± 0.000084	0.00062 ± 0.000067	0.00062 ± 0.000069
AUC of the power spectrum of the LFP (AUC)	0.03 ± 0.003	0.06 ± 0.008*	0.05 ± 0.009
AUC of the coherence of the ECoG/EP spikes (AUC)	0.31 ± 0.04	0.46 ± 0.05	0.56 ± 0.07*
AUC of the coherence of the LFP/EP spikes (AUC)	0.29 ± 0.03	0.45 ± 0.06	0.51 ± 0.05*
AUC of the coherence of the ECoG/LFP spikes (AUC)	1.1 ± 0.11	1.4 ± 0.21	1.0 ± 0.12

*Note*: Values are expressed as mean ± S.E.M. For the oscillatory and synchronization parameters (power spectra and coherences), AUC values were calculated. Abbreviations: CV, coefficient of variation; EcoG, electrocorticogram; LFP, local field potential

* *P* < .05 versus sham (one‐way ANOVA followed by Bonferroni's post hoc test).

^$^ *P* < .05 versus sham (Fisher's exact test).

### 3.2 Effect of 5‐HT_1A_ receptor agonists on entopeduncular nucleus neuron activity

Systemic administration of buspirone significantly inhibited the firing rate of entopeduncular nucleus neurons in the sham and 6‐OHDA/l‐DOPA groups (Figure 1c,d). However, this inhibition was two to three times more pronounced in the sham than in the 6‐OHDA/l‐DOPA animals (reduction of firing rate with respect to the basal values obtained after 5 mg·kg^−1^ buspirone for sham: 94.2 ± 2.9% and 6‐OHDA/l‐DOPA: 39.7 ± 14.5%). The inhibitory effect of systemic buspirone was lost in the 6‐OHDA group. In all cases, this effect was reversed by systemic administration of the 5‐HT_1A_ receptor antagonist, WAY‐100635 (0.5 mg·kg^−1^, i.v.). To further prove the contribution of 5‐HT_1A_ receptors to the observed effect, WAY‐100635 was applied before buspirone. In this case, the effect of buspirone in the sham and 6‐OHDA/l‐DOPA groups was blocked. In addition, no effect on the firing parameters of entopeduncular nucleus neurons was observed after WAY‐100635 administration (data not shown). For the firing pattern, buspirone significantly increased the coefficient of variation only in the sham group. Finally, buspirone increased the number of bursty neurons in the sham group, while it slightly decreased this parameter at small doses in the 6‐OHDA and 6‐OHDA/l‐DOPA groups; an increase or no change was observed at high doses (Figure 1e). Intraburst parameters were similar in all the groups (Table S2). To further understand the mechanism of buspirone, we performed local administrations in the entopeduncular nucleus and measured neuron firing activity. In all the experimental groups (*n* = 5 per group), local administration of buspirone (0.25–2 nM) caused a marked dose‐dependent inhibition of neuronal activity with a maximal significant reduction of the firing rate of approximately 70% of the basal value (Figure 1f). However, this inhibitory effect was similar among the groups.

We also investigated the effect of the systemic administration of the full 5‐HT_1A_ receptor agonist 8‐OH‐DPAT (from 20 μg·kg^−1^ to 160 μg·kg^−1^; i.v.) on entopeduncular nucleus activity in the sham, 6‐OHDA‐lesioned and 6‐OHDA/l‐DOPA animals. Drug administration caused a significant dose‐dependent inhibition of the entopeduncular nucleus neuron firing, which was significant for the highest doses in the sham and 6‐OHDA/l‐DOPA groups (Figure 1g). This effect was twice more pronounced in the sham group than in the 6‐OHDA/l‐DOPA group (reduction of firing rate with respect to the basal values obtained after 80 μg·kg^−1^ 8‐OH‐DPAT for sham:‐ 63.7 ± 12.1% and 6‐OHDA/l‐DOPA:‐ 27.2 ± 15.9%). The administration of WAY‐100635 (0.5–1 mg·kg^−1^, i.v.) reversed the inhibitory effect of 8‐OH‐DPAT. Once again, no inhibitory effect was observed in the 6‐OHDA group. On the other hand, 8‐OH‐DPAT administration caused a very mild increase in coefficient of variation at a dose of 40 μg·kg^−1^ in the 6‐OHDA/l‐DOPA group. Low doses of 8‐OH‐DPAT decreased the number of bursty neurons in the three experimental groups, while high doses increased it; however, the highest effect was observed in the sham group (Figure 1h). 8‐OH‐DPAT administration did not modify the intraburst parameters (Table S3).

Finally, we investigated the effect of the systemic administration of buspirone or 8‐OH‐DPAT on low oscillatory activity and synchronization. Buspirone administration significantly reduced the AUC of the electrocorticogram power spectra in the sham group (Figure 2b), while high doses significantly enhanced the oscillatory activity of local field potential‐entopeduncular nucleus only in the 6‐OHDA/l‐DOPA group (Figure 2c). For the effect on synchronization, buspirone did not modify coherence in any of the analysed situations (Figure 2d); minimal differences were observed between the 6‐OHDA/l‐DOPA and 6‐OHDA groups for the coherence between the local field potential and entopeduncular nucleus spikes (Figure 2e). 8‐OH‐DPAT administration did not modify the AUC of the electrocorticogram power spectrum (Figure 2f), but it significantly reduced the AUC values of the local field potential power spectrum in the 6‐OHDA/l‐DOPA group (Figure 2g). Coherence analysis revealed that 8‐OH‐DPAT did not cause any effect on the synchronization between electrocorticogram and entopeduncular nucleus or electrocorticogram and local field potential spikes (Figure 2h). However, it significantly reduced the AUC values of the coherence between the local field potential and entopeduncular nucleus spikes in the 6‐OHDA/l‐DOPA group (Figure 2i). Neither of the tested drugs modified the AUC values of coherence between the local field potential and electrocorticogram (data not shown).

### 3.3 Integrated optical densitometry for SERT and 5‐HT_1A_ receptor immunostaining in the basal ganglia and dorsal raphé nuclei

**FIGURE 3:**
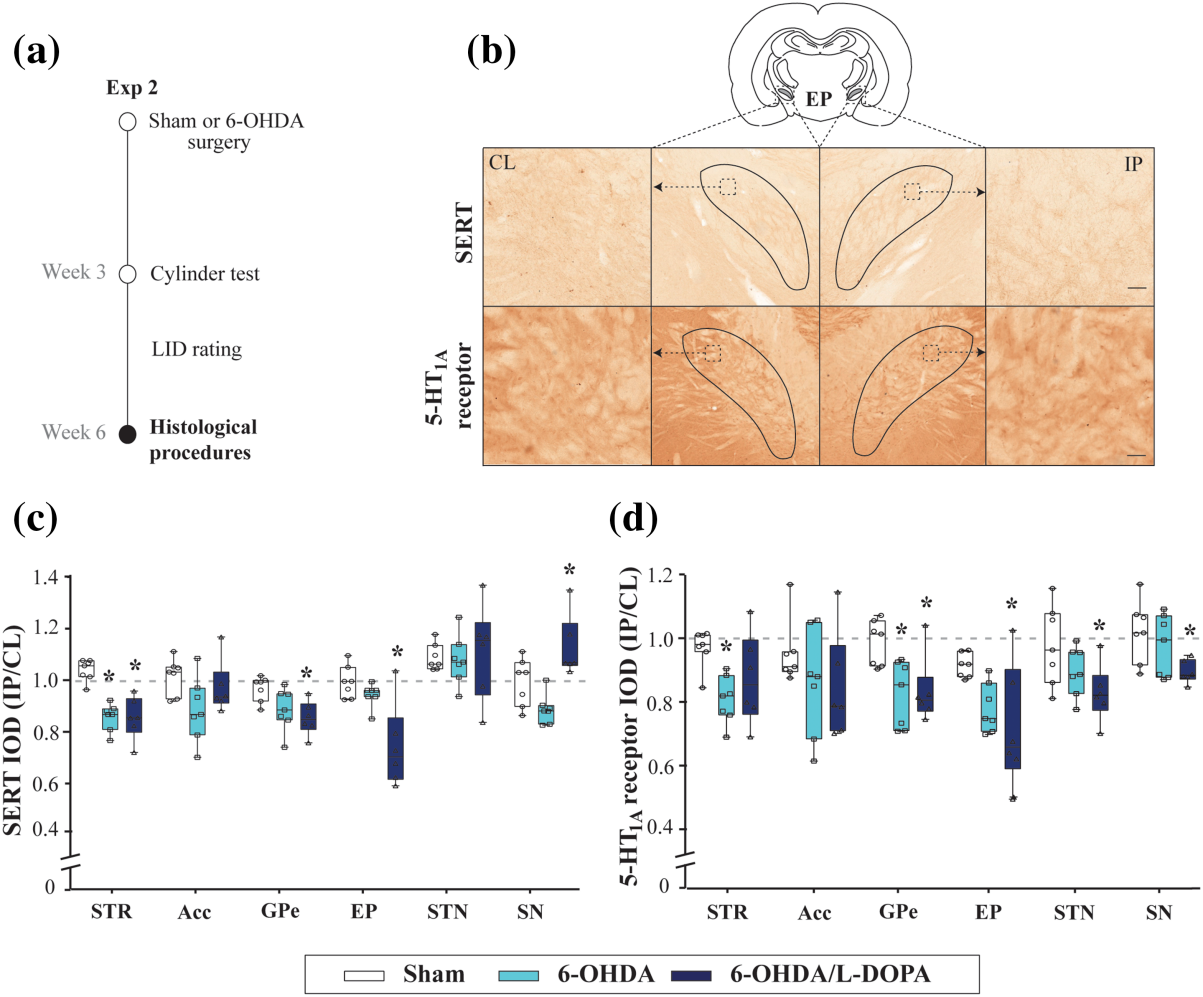
5‐HT_1A_ receptor and SERT immunoreactivity expression in the basal ganglia. (a) Design of experiment. The rats received 6‐OHDA or vehicle injection into the medial forebrain bundle and the accuracy of the lesion was screened using the cylinder test 3 weeks later. Six weeks after the 6‐OHDA lesion, 5‐HT_1A_ receptor and SERT immunohistochemistry were performed. (b) Representative coronal sections of the entopeduncular nucleus with the delimited regions from ipsilateral (IL) and contralateral (CL) hemispheres adapted from Paxinos and Watson (1997). The example corresponds to a sham animal. (c, d) Box and Whiskers representing the median and min to max values of IOD ratio of SERT (c) and 5‐HT_1A_ receptor immunoreactivity (d), from sham (*n* = 7), 6‐OHDA‐lesioned (*n* = 7) and 6‐OHDA/l‐DOPA (*n* = 6) groups. Note a general reduction in the expression of both markers especially in the 6‐OHDA/l‐DOPA group. STR: striatum; Acc: nucleus accumbens; GPe: external globus pallidus; EP: entopeduncular nucleus; STN: subthalamic nucleus; SN: substantia nigra; respectively. *P < .05 versus sham group (one‐way ANOVA followed by Bonferroni's post hoc test)

**FIGURE 4:**
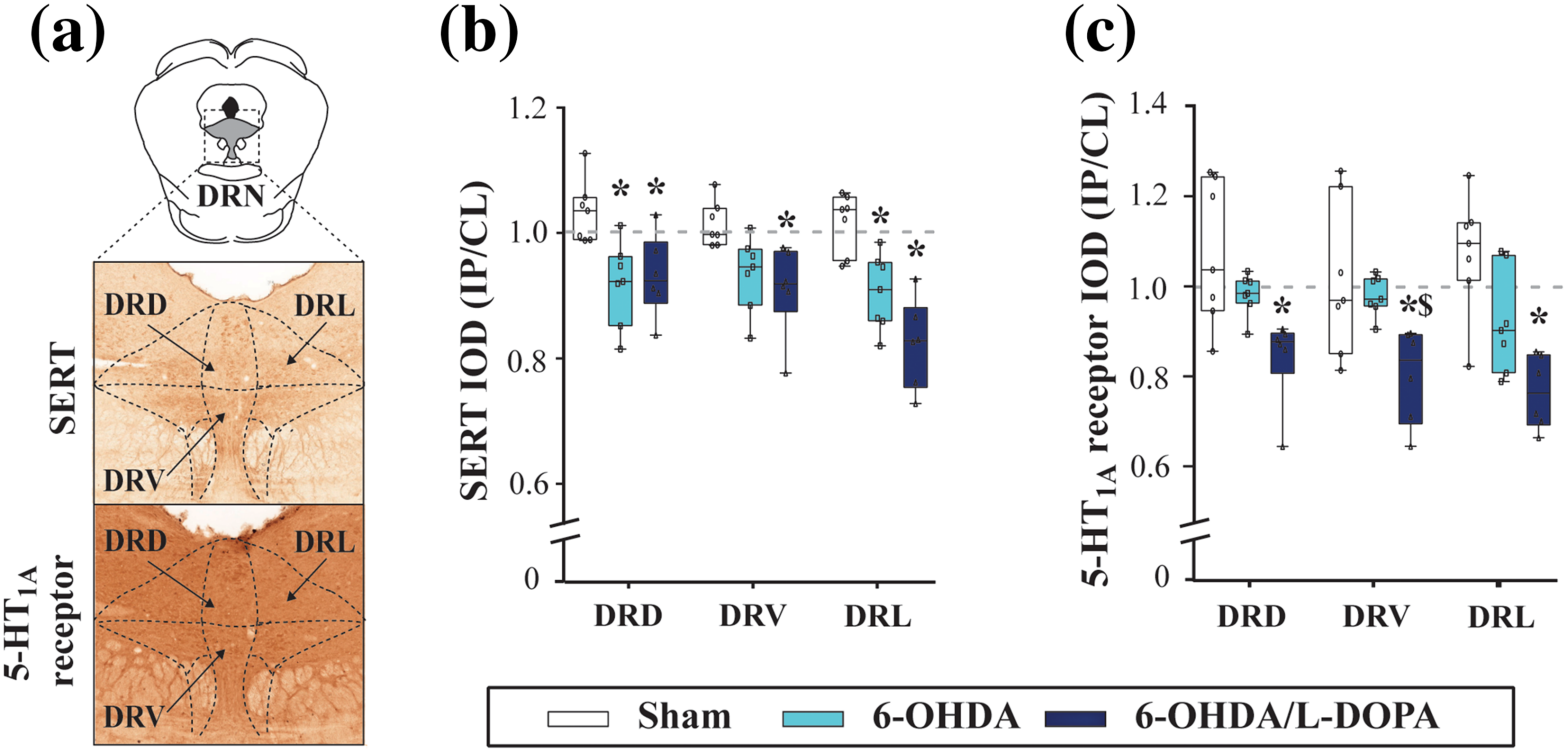
5‐HT_1A_ receptor and SERT immunoreactivity expression in the dorsal raphé nucleus (DRN). (a) Representative coronal sections of the DRN with the delimited regions from ipsilateral (IL) and contralateral (CL) hemispheres adapted from Paxinos and Watson (1997). The example corresponds to a sham animal. (c, d) Box and Whiskers representing the median and min to max values of integrated optical densitometry (IOD) ratio of SERT (c) and 5‐HT_1A_ receptor immunoreactivity (d) from sham (*n* = 7), 6‐OHDA‐lesioned (*n* = 7), and 6‐OHDA/l‐DOPA (*n* = 6) groups. Note a general reduction in the expression of both markers especially in the 6‐OHDA/l‐DOPA group. DRD, DRV and DRL: dorsal, ventral and lateral regions of the DRN, respectively. *P < .05 versus sham group and ^$^ *P* < .05 versus 6‐OHDA group (one‐way ANOVA followed by Bonferroni's post hoc test)

To better understand the electrophysiological results and following the same experimental design, we evaluated SERT and 5‐HT_1A_ receptor immunoreactivities in the basal ganglia and DRN in the three experimental groups (sham, *n* = 7; 6‐OHDA, *n* = 7; 6‐OHDA/l‐DOPA, *n* = 6 animals) (Figure 3a,b). We analysed the immunoreactivities in the dorsal, ventral and lateral regions of the DRN (DRD, DRV and DRL, respectively) (Figure 4a). In the 6‐OHDA group, the average IOD for SERT immunoreactivity (Figures 3c and 4b) was unmodified, overall, but slightly reduced in the dorsal striatum or DRD. This reduction was more consistently observed in most areas measured, striatum, external globus pallidus, entopeduncular nucleus, DRD, DRV and DRL in the 6‐OHDA/l‐DOPA rats. Surprisingly, IOD for SERT immunoreactivity in the substantia nigra was significantly increased in this latter group.

As for 5‐HT_1A_ receptor immunoreactivity (Figure 3d and 4c), a slight reduction in IOD was found in the striatum and external globus pallidus of 6‐OHDA animals. Similar to SERT immunoreactivity, a reduction of IOD of 5‐HT_1A_ receptor immunoreactivity was observed in the external globus pallidus, entopeduncular nucleus, subthalamic nucleus, substantia nigra, DRD, DRV and DRL in the 6‐OHDA/l‐DOPA group. The IOD of the immunoreactivity of these two 5‐HT markers was not modified in the nucleus accumbens in any of the studied situations.

### 3.4 Effect of buspirone on opto‐stimulation entopeduncular response

**FIGURE 5:**
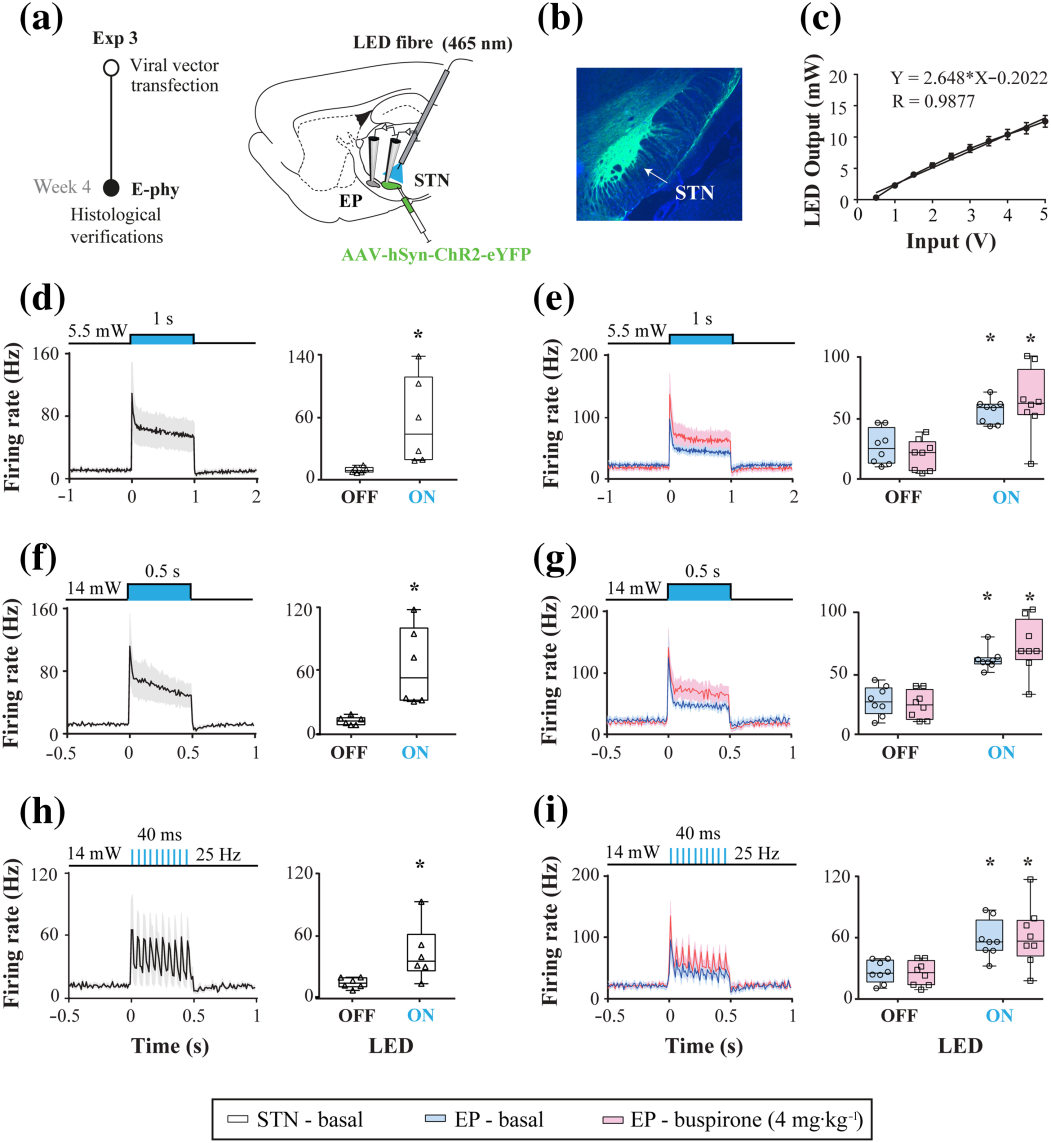
Entopeduncular nucleus (EP) responses after subthalamic nucleus (STN) opto‐stimulation and buspirone administration. (a) Experimental design and a representative parasagittal section of a rat brain showing the viral vector AAV5‐hSyn‐hChR2(H134R)‐EYFP injection into the STN. Four weeks after the viral injection, the LED fibre was positioned within the STN and STN or EP single‐unit extracellular electrophysiology (E‐phy) was performed and analysed during optostimulation. (b) Coronal section showing viral transfection in the STN of the injected hemisphere. (c) Nonlinear standard curve generated from the calibration of LED illumination at the beginning of the experiments. (d, e) Effect of light pulse (1 s, ~5.5 mW) on the firing rate of STN neurons (d) and EP neurons (e). Peristimulus histograms were constructed to show the STN firing rate before, during, and after local optical stimulation with 465‐nm blue‐light pulses. (f, g) Same as d and e for light pulses of 0.5 s, ~14 mW. (h, i) Same as d and e for trains of stimulations at 25 Hz, during 40 ms and ~14 mW. Note that STN opto‐stimulation increased the EP neuron firing frequency, and buspirone administration did not modify the type of response in the EP induced by STN opto‐stimulation. Box and Whiskers with averaged plots represent the median and min to max values of firing rate value of STN (white) and EP neurons (blue), *n* = 6 and 8, respectively. Buspirone (4 mg·kg^−1^, i.p.) administration is highlighted in pink. *P < .05 versus light off (two‐tailed paired Student's *t*‐test or RM two‐way ANOVA followed by Bonferroni's post hoc test)

Finally, we carried out opto‐stimulation of the subthalamic nucleus and simultaneous single‐unit extracellular recordings of subthalamic nucleus or entopeduncular nucleus neurons in control animals 4 weeks after the viral injection. Only rats that showed a stimulatory response in most of the recorded neurons were included in this study. A total of 40 subthalamic nucleus neurons were recorded in six animals (five to nine neurons per animal) before and during opto‐stimulation using three different protocols:‐ continuous pulses (1‐s duration at ~5.5‐mW intensity or 0.5‐s duration at ~14‐mW intensity) and trains of light pulses (25‐Hz frequency, 0.5‐s duration at ~14‐mW intensity) (Figure 5d,f,h). For all the protocols, opto‐stimulation significantly excited 35 subthalamic nucleus neurons (87.5%). On average, the subthalamic nucleus neuron firing rate was significantly higher than the basal value (Figure 5d), almost six times during the first protocol, almost five times during the second (Figure 5f) and almost three times during the third (Figure 5h).

Next, we carried out single‐unit extracellular recordings of entopeduncular nucleus neurons from eight animals (three to seven neurons per animal), while the optic fibre was positioned in the subthalamic nucleus. subthalamic nucleus opto‐stimulation increased the entopeduncular nucleus firing rate in 26 of the 36 recorded cells (72.2%) under basal conditions and 19 of the 24 recorded neurons (79.2%) after buspirone administration. Regardless of the applied protocol, entopeduncular nucleus firing activity increased during subthalamic nucleus stimulation. However, buspirone administration (4 mg·kg^−1^, i.p.) did not modify the intensity of the response (Figure 5e–i).

## 4 DISCUSSION AND CONCLUSIONS

This study extends our previous finding regarding how buspirone affects basal ganglia nuclei showing that, (1) 5‐HT_1A_ receptor activation elicits different effects on the entopeduncular nucleus electrophysiological properties depending on the integrity of the nigrostriatal pathway and the location of the receptor, (2) IODs for SERT and 5‐HT_1A_ receptor immunoreactivity are differently regulated by 6‐OHDA‐lesion along the basal ganglia and DRN and (3), buspirone does not alter the relationship between subthalamic nucleus and entopeduncular nucleus neuronal activity.

As we and other authors have previously shown, there was an increase in burst activity in entopeduncular nucleus neurons from 6‐OHDA‐lesioned rats and an enhancement in the firing rate after prolonged treatment with l‐DOPA (Aristieta et al., 2019; Jin et al., 2016). In addition, the effect of systemic buspirone was significantly different. Thus, buspirone mainly reduced the firing rate in the sham group and the burst activity in the 6‐OHDA‐lesioned groups. A similar effect on firing rate but not in burst activity was observed for 5‐HT_1A_ receptor activation by 8‐OH‐DPAT. Both firing rate and firing patterns of neurons from the basal ganglia are altered in animal models of Parkinson's disease (McGregor & Nelson, 2019). In particular, the loss of dopamine in the parkinsonian condition leads to an increase in subthalamic nucleus activity, producing augmented excitatory input to the basal ganglia output structures (entopeduncular nucleus and substantia nigra reticulata) and contributing to the hyperactivity of these nuclei, which may underlie motor slowing (Alexander et al., 1986). Buspirone has, however, little effect on low‐frequency oscillation and synchronization and the local effects of 5‐HT_1A_ receptor activation were not influenced by dopamine. Since buspirone shows potent anti‐dyskinetic properties in behavioural studies (see Section 1), the present data together with our previous results (Sagarduy et al., 2016; Vegas‐Suárez et al., 2020) highlight the importance of firing patterns rather than oscillations in dopamine‐depleted conditions when testing the anti‐parkinsonian/anti‐dyskinetic effect of a drug.

Buspirone acts as an antagonist at D_2_ and D_3_ dopamine receptors (Bergman et al., 2013; Dhavalshankh et al., 2007; McMillen & McDonald, 1983). In addition, all dopamine receptor subtypes are detected in the entopeduncular nucleus (Lavian et al., 2017; Lavian et al., 2018) suggesting that buspirone's local effect could be mediated by blockade ofdopamine receptors. This is however unlikely, since in that case, buspirone's local administration would have produced no effect or an increment in neuronal activity. This is based on functional studies, which have shown that dopamine agonists *in vivo* and *in vitro* reduce the firing activity of entopeduncular nucleus neurons (Boraud et al., 1998; Lacombe et al., 2009; Lavian et al., 2017; Papa et al., 1999; Périer et al., 2003; Ruskin et al., 2002). In addition, when buspirone was applied systemically, the effects we observed were reversed by 5‐HT_1A_ receptor antagonism, suggesting a selective 5‐HT_1A_ receptor‐mediated effect of this drug. On the other hand, our results stress that buspirone's effect in the entopeduncular nucleus involves direct and indirect mechanisms since its local effect is greater than the systemic effect. This effect is affected by dopamine depletion, probably due to 5‐HT neurotransmission changes outside the entopeduncular nucleus or in the 5‐HT input to the entopeduncular nucleus. Our results show a slight reduction in IOD of SERT immunoreactivity in the dorsal striatum after the 6‐OHDA‐lesion and l‐DOPA treatment in the striatum, external globus pallidus and entopeduncular nucleus; SERT expression increased in the substantia nigra reticulata. Published results show that striatal SERT expression in parkinsonian rats chronically treated with l‐DOPA is up‐regulated (Rylander et al., 2010; Tronci et al., 2017), not modified (Prinz et al., 2013) or even decreased (Nevalainen et al., 2014; Walker et al., 2019). In support of our results, reduced SERT‐binding has also been found in the striatum and external globus pallidus from patients with Parkinson's disease (Albin et al., 2008; Chinaglia et al., 1993; Kish et al., 2007). Similar to the present results, in our previous study we found that the IOD for SERT immunoreactivity was unchanged and increased in the substantia nigra reticulata after 6‐OHDA lesions with and without l‐DOPA treatment, respectively (Vegas‐Suárez et al., 2020). On the other hand, we observed a discrete reduction in the IOD of SERT immunoreactivity in the DRN (the origin of the main 5‐HT innervation to the basal ganglia, Benarroch, 2009; Huang et al., 2019) after the 6‐OHDA lesion and l‐DOPA treatment, as observed in patients with early and advanced Parkinson's disease (Chinaglia et al., 1993; Halliday et al., 1990; Kish et al., 2007; Politis et al., 2014). The IOD of 5‐HT_1A_ receptor immunoreactivity was slightly but significantly decreased after l‐DOPA treatment in the external globus pallidus, entopeduncular nucleus, subthalamic nucleus and substantia nigra. Although no changes in mRNA levels and protein expression were found in the striatum of the 6‐OHDA‐lesioned rats (Numan et al., 1995; Radja et al., 1993), 5‐HT_1A_ receptor expression is decreased in the caudate nucleus and cingulate cortex of patients with Parkinson's disease (Ballanger et al., 2012) but not in the external globus pallidus and substantia nigra (Huot et al., 2012). Regarding the different regions of the DRN (DRV, DRD and DRL), we found decreased IOD of 5‐HT_1A_ receptor immunoreactivity in the 6‐OHDA/l‐DOPA group, as previously described in rats (Hou et al., 2012), monkeys (Frechilla et al., 2001) and patients with Parkinson's disease (Braak et al., 2003; Doder, Rabiner et al., 2003; Halliday et al., 1990). Overall, our results suggest that the 5‐HT system undergoes degeneration after the 6‐OHDA lesion, which is worsened by l‐DOPA treatment in agreement with the proposed l‐DOPA neurotoxic effect on 5‐HT neurons (Stansley & Yamamoto, 2014). Both IODs for SERT and of 5‐HT_1A_ receptor immunoreactivity were reduced suggesting that these receptors are located on 5‐HT terminals. However, 5‐HT_1A_ receptors postsynaptically located on the entopeduncular nucleus seem to be less affected by the 6‐OHDA lesions and l‐DOPA treatment. This may explain why the systemic 5‐HT_1A_ receptor agonist effect is altered while the local effect remains unchanged. It is important to point out that the quantitative estimation of the changes in SERT and 5‐HT_1A_ receptors expression using immunohistochemistry should be confirmed with truly quantitative methods. such as quantitative autoradiography.

The basal ganglia nuclei, including the subthalamic nucleus, contain 5‐HT_1A_ receptors and receive intense 5‐HT innervation (Miguelez et al., 2014). We used optogenetic stimulation of the subthalamic nucleus to test whether 5‐HT_1A_ receptor activation could alter the subthalamic nucleus‐entopeduncular nucleus relationship when the subthalamic nucleus is hyperactive after dopamine loss (Aristieta et al., 2012, 2019). First, we found that the direct application of light into the subthalamic nucleus previously transfected with AAV5‐hSyn‐hChR2‐(H134R)‐EYFP increased the firing rate of most but not all subthalamic nucleus recorded neurons. This heterogeneity in the subthalamic nucleus neuronal response has been described by other authors, who attribute it to the activation of inhibitory neurons within the nucleus that suppresses the activities of the recorded neurons (Yu et al., 2020). In addition, we observed that continuous blue‐light pulses lasting for 1 s with a 5.5‐mW intensity or 0.5 s with 14 mV caused a higher excitation of subthalamic nucleus and entopeduncular nucleus neurons than the trains of 25‐Hz pulses with a duration of 0.5 s and intensity of approximately 14 mW, suggesting the presence of neuron response saturation, as previously reported (Herman et al., 2014; Lin, 2011; Lin et al., 2009). Subthalamic nucleus opto‐stimulation also enhanced the activity of most recorded entopeduncular nucleus neurons and the effect was not related to the intensity or duration of the subthalamic nucleus stimulation. These results are consistent with previous findings that showed a significant correlation between the firing rates of entopeduncular nucleus and subthalamic nucleus neurons when both nuclei were hyperactive (Aristieta et al., 2019). There was an increase in entopeduncular nucleus activity following subthalamic nucleus drug stimulation (Robledo et al., 1988) and an increase in extracellular glutamate levels in the entopeduncular nucleus induced by subthalamic nucleus stimulation (Windels et al., 2000). Note that the present study does not replicate the conditions of those experiments that apply electrical subthalamic nucleus‐dyskinesia causing an inhibition (Benazzouz et al., 1995) or excitation of the entopeduncular nucleus (Hashimoto et al., 2003). In these studies, the effect of subthalamic nucleus‐deep brain stimulation may result in the suppression of neuronal activity due to depolarization blockade.

Next, we investigated whether buspirone modified the effect of subthalamic nucleus opto‐stimulation on entopeduncular nucleus because buspirone reduces the subthalamic nucleus neuron activity under control conditions but not after the 6‐OHDA lesion when the subthalamic nucleus is hyperactive (Sagarduy et al., 2016). In line with this, our results show no effect of buspirone on entopeduncular nucleus stimulation induced by subthalamic nucleus optogenetic activation, suggesting that subthalamic nucleus hyperactivity may dampen buspirone's effect on this basal ganglia output nucleus. It is important to point out that several studies have shown that high frequency stimulation of the subthalamic nucleus induces inhibition of raphé 5‐HT neuronal activity (Kocabicak et al., 2015; Temel et al., 2007) and suppression of subthalamic nucleus evoked neuronal activity (Shehab et al., 2018)**.** However, the low intensity and brief stimulation we applied caused stimulation of the subthalamic nucleus. Altogether, our results indicate that when the subthalamic nucleus is hyperactive due to dopamine loss or opto‐stimulation, buspirone has little effect on the entopeduncular nucleus. Whether this reduction in the buspirone effect has any clinical relevance is difficult to elucidate. Interestingly, there is a phase I clinical trial (NCT02589340) that aims to evaluate the efficacy of combination therapy using buspirone and amantadine in reducing l‐DOPA induced dyskinesia in patients with Parkinson's disease (McFarthing et al., 2019). Electrophysiological results have shown that amantadine increases subthalamic nucleus neuron activity (Allers et al., 2005).

The incidence of Parkinson's disease is progressively increasing (Tysnes & Storstein, 2017) and there is not yet a way to prevent l‐DOPA‐induced dyskinesia (Cenci et al., 2020). Nowadays, one of the most efficacious treatment when l‐DOPA loses efficacy or causes severe adverse effects is the deep brain stimulation, an invasiveness technique with several limitations. There is therefore a necessity of developing new drugs for treating l‐DOPA‐induced dyskinesia. To do that, the target and responses that may help to test new drugs in preclinical studies should be characterized first. Our present findings are in line with those of previous studies (Sagarduy et al., 2016; Vegas‐Suárez et al., 2020), showing that the effect of 5‐HT_1A_ receptor activation is damped by dopamine loss. Furthermore, buspirone in 6‐OHDA‐lesioned rats mainly affects entopeduncular nucleus‐burst activity and it does not alter subthalamic nucleus control over the entopeduncular nucleus. Overall, our results provide knowledge about the 5‐HT_1A_ receptor‐effects of a potential anti‐dyskinetic drug, buspirone, on the entopeduncular nucleus of an experimental model of Parkinson's disease prolonged treated with l‐DOPA.

## AUTHOR CONTRIBUTIONS

L.U. and C.M. conceived the study, designed the experiments and drafted the final version of the manuscript. S.V.S. performed all the experiments, carried out data quantification and analysis, prepared the figures and contributed to the first draft. A. A. and C. M. collaborated in the optogenetic experiments. C.R. and H.B. contributed to the immunostaining experiment design. C.M. revised the data analysis. S.V.S., C.M., J.V.L., A.S. and L.U. interpreted the results. All authors reviewed and edited the manuscript.

## CONFLICT OF INTEREST

The authors declare no conflicts of interest.

## DECLARATION OF TRANSPARENCY AND SCIENTIFIC RIGOUR

This Declaration acknowledges that this paper adheres to the principles for transparent reporting and scientific rigour of preclinical research as stated in the *BJP* guidelines for Design & Analysis, Immunoblotting and Immunochemistry and Animal Experimentation, and as recommended by funding agencies, publishers, and other organisations engaged in supporting the research.

## Supporting information


**Table S1.** Supporting information

**Table S2.** Effect of systemic administration of buspirone on firing properties of entopeduncular neurons

**Table S3.** Effect of 8‐OH‐DPAT on firing properties of entopeduncular nucleus (EP) neurons

**Figure S1.** **Behavioural tests.** (A) Motor asymmetry was evaluated comparing the use of the forelimb contralateral (CL) and ipsilateral (IL) to the lesion in the cylinder test**,** data are expressed as the mean value of the percentage of ipsilateral or contralateral touches divided into the total number represented as Box and Whiskers representing the median and Min to Max values (*P < .05, two‐tailed paired Student's *t*‐test). Note that 6‐OHDA‐lesioned animals preferably use the ipsilateral to the lesion. (B) Evolution of dyskinesia scores showing the time course of abnormal involuntary movements (AIMs) scores for axial, limb and orolingual ratings and (C) locomotive score, on the last session after l‐DOPA chronic treatment. All the 6‐OHDA/l‐DOPA animals enrolled in this study developed severe l‐DOPA‐induced dyskinesia (LID). Results are expressed as mean ± S.E.M.

## Data Availability

The data that support the findings of this study are available from the corresponding author upon reasonable request.
