# Supplementary material for: The effect of 5‐HT1A receptor agonists on the entopeduncular nucleus is modified in 6‐hydroxydopamine‐lesioned rats
Source: Br J Pharmacol. 2021 May 6;178(12):2516–32. doi: 10.1111/bph.15437 (PMC8252460; doi:10.1111/bph.15437)
Supplement: Supplementary file 1 — Table S1. Supporting information [file BPH-178-2516-s001.pdf]

Electrophysiology	Sham<br><br>Mean + SD	6-OHDA<br><br>Mean + SD	6-OHDA/<br>L-DOPA<br>Mean + SD	One-way ANOVA<br><br>F(DFn, DFd)	P value
**Firing rate (Hz)**	17.4 ± 6.4	24.6 ±11.5	27.4 ± 11.6	$F_{(2, 45)} = 4.153$	$p < 0.05$
**Coefficient of Variation (%)**	46.7 ± 23.2	54.9 ± 37.6	82.4 ± 46.5	$F_{(2, 45)} = 4.044$	$p < 0.05$
**ECoG Power spectrum (AUC values)**	0.0005 ± 0.0003	0.0006 ± 0.0003	0.0006 ± 0.0003	$F_{(2, 45)} = 0.67$	n.s.
**LFP Power spectrum (AUC values)**	0.03 ± 0.01	0.06 ± 0.03	0.053 ± 0.04	$F_{(2, 45)} = 3.46$	$p < 0.05$
**Coherence EcoG-EP spikes (AUC values)**	0.31 ± 0.16	0.46 ± 0.22	0.56 ± 0.32	$F_{(2, 45)} = 4.11$	$p < 0.05$
**Coherence LFP-EP spikes (AUC values)**	0.29 ± 0.12	0.45 ± 0.25	0.51 ± 0.22	$F_{(2, 45)} = 4.73$	$p < 0.05$
**Coherence ECoG-LFP (AUC values)**	1.16 ± 0.44	1.4 ± 0.84	1.0 ± 0.51	$F_{(2, 45)} = 1.71$	n.s.

SERT immunohistochemistry	Sham<br>Mean + SD	6-OHDA<br>Mean + SD	6-OHDA/L-DOPA<br>Mean + SD	One-way ANOVA<br>F(DFn, DFd)	P value
**Striatum**	1.04 ± 0.04	0.86 ± 0.05	0.86 ± 0.08	$F_{(2,17)} = 20.94$	$p < 0.05$
**Acc**	1.01 ± 0.07	0.89 ± 0.13	0.97 ± 0.10	$F_{(2,17)} = 2.97$	n.s.
**GP**	0.97 ± 0.05	0.89 ± 0.08	0.85 ± 0.07	$F_{(2,17)} = 5.04$	$p < 0.05$
**EP**	0.99 ± 0.06	0.94 ± 0.05	0.74 ± 0.16	$F_{(2,17)} = 11.42$	$p < 0.05$
**STN**	1.09 ± 0.05	1.08 ± 0.09	1.11 ± 0.18	$F_{(2,17)} = 0.13$	n.s.
**SN**	0.99 ± 0.09	0.89 ± 0.06	1.13 ± 0.12	$F_{(2,17)} = 11.01$	$p < 0.05$
**DRD**	1.04 ± 0.05	0.91 ± 0.07	0.93 ± 0.07	$F_{(2,17)} = 7.40$	$p < 0.05$
**DRV**	1.01 ± 0.04	0.93 ± 0.06	0.90 ± 0.07	$F_{(2,17)} = 6.06$	$p < 0.05$
**DRL**	1.02 ± 0.05	0.90 ± 0.06	0.81 ± 0.07	$F_{(2,17)} = 17.52$	$p < 0.05$

SERT immunohistochemistry IOD (CL hemisphere)	Sham<br>Mean + SD	6-OHDA<br>Mean + SD	6-OHDA/L-DOPA<br>Mean + SD	One-way ANOVA<br>F(DFn, DFd)	P value
**Striatum**	69.97 ± 9.67	73.81 ± 11.95	66.17 ± 6.49	$F_{(2,17)} = 0.98$	n.s.
**Acc**	33.60 ± 10.61	37.71 ± 7.24	29.67 ± 4.80	$F_{(2,17)} = 1.61$	n.s.
**GP**	69.38 ± 11.85	73.23 ± 10.56	68.50 ± 6.63	$F_{(2,17)} = 0.42$	n.s.

**EP**	69.53 ± 16.78	69.68 ± 9.80	62.50 ± 9.67	$F_{(2,17)} = 0.66$	n.s.
**STN**	62.42 ± 14.21	66.61 ± 8.49	59.67 ± 14.56	$F_{(2,17)} = 0.50$	n.s.
**SN**	70.95 ± 23.26	70.39 ± 20.96	54.00 ± 24.35	$F_{(2,17)} = 1.28$	n.s.
**5-HT_{1A} receptor immunohistochemistry IOD (IP/CL)**	**Sham Mean + SD**	**6-OHDA Mean + SD**	**6-OHDA/L-DOPA Mean + SD**	**One-way ANOVA F(DFn, DFd)**	**P value**
**Striatum**	0.97 ± 0.07	0.81 ± 0.03	0.87 ± 0.14	$F_{(2,17)} = 5.13$	$p < 0.05$
**Acc**	0.95 ± 0.10	0.86 ± 0.16	0.84 ± 0.16	$F_{(2,17)} = 1.06$	n.s.
**GP**	0.99 ± 0.07	0.82 ± 0.10	0.83 ± 0.10	$F_{(2,17)} = 6.33$	$p < 0.05$
**EP**	0.91 ± 0.03	0.78 ± 0.07	0.72 ± 0.19	$F_{(2,17)} = 4.87$	$p < 0.05$
**STN**	0.98 ± 0.12	0.90 ± 0.07	0.83 ± 0.03	$F_{(2,17)} = 3.64$	$p < 0.05$
**SN**	1.02 ± 0.10	0.98 ± 0.10	0.9 ± 0.04	$F_{(2,17)} = 3.70$	$p < 0.05$
**DRD**	1.06 ± 0.16	0.97 ± 0.05	0.84 ± 0.10	$F_{(2,17)} = 6.85$	$p < 0.05$
**DRV**	1.01 ± 0.17	0.97 ± 0.04	0.80 ± 0.10	$F_{(2,17)} = 5.69$	$p < 0.05$
**DRL**	1.06 ± 0.13	0.91 ± 0.11	0.76 ± 0.08	$F_{(2,17)} = 5.13$	$p < 0.05$
**5-HT_{1A} receptor immunohistochemistry IOD (CL hemisphere)**	**Sham Mean + SD**	**6-OHDA Mean + SD**	**6-OHDA/L-DOPA Mean + SD**	**One-way ANOVA F(DFn, DFd)**	**P value**

**Striatum**	48.25 ± 10.98	46.45 ± 12.99	38.09 ± 11.64	$F_{(2,17)} = 1.31$	n.s.
**Acc**	47.97 ± 20.50	44.38 ± 7.87	34.02 ± 13.31	$F_{(2,17)} = 1.40$	n.s.
**GP**	31.49 ± 6.92	39.91 ± 17.26	28.73 ± 11.10	$F_{(2,17)} = 1.41$	n.s.
**EP**	36.92 ± 9.27	49.40 ± 20.71	36.47 ± 12.81	$F_{(2,17)} = 1.59$	n.s.
**STN**	70.64 ± 49.34	40.30 ± 25.68	56.96 ± 24.36	$F_{(2,17)} = 1.28$	n.s.
**SN**	90.22 ± 45.68	97.26 ± 40.73	69.61 ± 17.80	$F_{(2,17)} = 0.93$	n.s.

Local administration of buspirone						
**Firing Rate**	**Sham**<br>**Mean ± SD**	**6-OHDA**<br>**Mean ± SD**	**6-OHDA/L-DOPA**<br>**Mean ± SD**	**Two WAY ANOVA**		
**Basal**	15.9 ± 5.3	21.3 ± 4.6	20.1 ± 1.1	**Interaction**	$F_{(8, 48)} = 1.74$	n.s.
**Busp 0.25**	13.7 ± 4.5	16.2 ± 3.6	12.3 ± 2.1	**Buspirone**	$F_{(4, 48)} = 54.72$	$p < 0.05$
**Busp 0.5**	13.3 ± 6.1	13.8 ± 2.8	9.7 ± 1.8	**Lesion**	$F_{(2, 12)} = 1.43$	n.s.
**Busp 1**	7.1 ± 4.0	9.1 ± 2.8	7.5 ± 1.6			
**Busp 2**	3.8 ± 2.6	7.8 ± 4.5	6.5 ± 4.6			

Firing Rate	Sham<br>Mean ± SD	6-OHDA<br>Mean ± SD	6-OHDA/<br>L-DOPA<br>Mean ± SD	Two WAY ANOVA		
**Basal**	14.6 ± 6.1	20.2 ± 7.5	26.7 ± 8.3	**Interaction**	$F_{(8, 56)} = 1.054$	$p < 0.05$
**Busp 0.6125**	7.2 ± 5.2	16.1 ± 4.0	21.5 ± 8.9	**Buspirone**	$F_{(4, 56)} = 15.45$	$p < 0.05$
**Busp 1.25**	2.7 ± 3.1	15.4 ± 3.5	17.1 ± 10.7	**Lesion**	$F_{(2, 14)} = 7.836$	$p < 0.05$
**Busp 2.5**	2.3 ± 3.2	15.1 ± 5.0	16.7 ± 10.9			
**Busp 5**	1.2 ± 1.3	15.2 ± 7.5	15.9 ± 9.5			
**Reversion with WAY-100635**	14.8 ± 4.2	10.8 ± 6.0	18.8 ± 12.3	**Interaction**	$F_{(2, 14)} = 1.52$	n.s.
				**Buspirone**	$F_{(1, 14)} = 5.82$	$P < 0.05$
				**Lesion**	$F(_{2, 14)} = 2.857$	n.s.

Coefficient of Variation	Sham<br>Mean ± SD	6-OHDA<br>Mean ± SD	6-OHDA/<br>L-DOPA<br>Mean ± SD	Two WAY ANOVA		
**Basal**	51.6 ± 22.2	42.2 ± 10.7	78.5 ± 45.2	**Interaction**	$F_{(8, 56)} = 1.819$	n.s.
**Busp 0.6125**	117.6 ± 100.6	49.0 ± 19.8	89.4 ± 90.9	**Buspirone**	$F_{(4, 56)} = 6.424$	$p < 0.05$
**Busp 1.25**	141.5 ± 106.5	48.0 ± 19.1	124.6 ± 123.7	**Lesion**	$F_{(2, 14)} = 1.\ 929$	n.s.

**Busp 2.5**	173.2 ± 129.7	48.3 ± 16.6	123.3 ± 112.1			
**Busp 5**	163.2 ± 103.4	54.5 ± 14.9	135.9 ± 115.0			
**Reversion with WAY-100635**	71.3 ± 23.37	43.15 ± 19.1	100.8 ± 46.8	**Interaction**	$F_{(2, 14)} = 0.878$	n.s.
				**Buspirone**	$F_{(1, 14)} = 4.953$	$p < 0.05$
				**Lesion**	$F_{(2, 14)} = 4.172$	$p < 0.05$

**% effect on ECoG**	**6-OHDA/L-DOPA Mean ± SD**	**Two WAY ANOVA**		
**Basal**	100	**Interaction**	$F_{(8, 56)} = 1.10$	n.s.
**Busp 0.6125**	97.3 ± 25.0	**Buspirone**	$F_{(4, 56)} = 3.30$	$p < 0.05$
**Busp 1.25**	101.3 ± 30.9	**Lesion**	$F_{(2, 14)} = 4.70$	$p < 0.05$
**Busp 2.5**	116.9 ± 30.5			
**Busp 5**	117.8 ± 34.1			

**% effect on LFP**	**6-OHDA/ L-DOPA Mean ± SD**	**Two WAY ANOVA**		

**Basal**	100	**Interaction**	$F_{(8, 56)} = 3.50$	$p < 0.05$
**Busp 0.6125**	83.28 ± 15.1	**Buspirone**	$F_{(4, 56)} = 6.04$	$p < 0.05$
**Busp 1.25**	65.7 ± 17.4	**Lesion**	$F_{(2, 14)} = 5.58$	$p < 0.05$
**Busp 2.5**	66.9 ± 16.2			
**Busp 5**	85.0 ± 53.3			
**% effect on coherence EcoG/EP spikes**	**6-OHDA/ L-DOPA Mean ± SD**	**Two WAY ANOVA**		
**Basal**	100	**Interaction**	$F_{(8, 56)} = 2.30$	$p < 0.05$
**Busp 0.6125**	97.3 ± 57.7	**Buspirone**	$F_{(4, 56)} = 1.43$	n.s.
**Busp 1.25**	102.5 ± 37.9	**Lesion**	$F_{(2, 14)} = 3.10$	n.s.
**Busp 2.5**	114.7 ± 50.5			
**Busp 5**	109.4 ± 55.5			
**% effect on coherence LFP/EP spikes**	**6-OHDA/ L-DOPA Mean ± SD**	**Two WAY ANOVA**		
**Basal**	100	**Interaction**	$F_{(8, 56)} = 0.92$	n.s.
**Busp 0.6125**	66.6 ± 36.4	**Buspirone**	$F_{(4, 56)} = 1.86$	n.s.

**Busp 1.25**	77.9 ± 51.2	**Lesion**	$F_{(2, 14)} = 0.29$	n.s.
**Busp 2.5**	73.4 ± 62.2			
**Busp 5**	91.3 ± 75.6			

**Systemic administration of 8-OH-DPAT**						
**Firing rate**	**Sham Mean ± SD**	**6-OHDA Mean ± SD**	**6-OHDA/L-DOPA Mean ± SD**	**Two WAY ANOVA**		
**Basal**	14.3 ± 5.8	26.4 ± 5.1	30.1 ± 13.3	**Interaction**	$F_{(8, 52)} = 2.30$	$p < 0.05$
**8-OH-DPAT 20**	11.4 ± 4.9	29.2 ± 7.9	29.5 ± 9.4	**Buspirone**	$F_{(4, 52)} = 3.28$	$p < 0.05$
**8-OH-DPAT 40**	8.6 ± 5.4	29.1 ± 8.8	26.2 ± 10.6	**Lesion**	$F_{(2, 13)} = 10.05$	$p < 0.05$
**8-OH-DPAT 80**	4.3 ± 3.1	30.4 ± 6.6	22.0 ± 13.6			
**8-OH-DPAT 160**	5.5 ± 2.8	30.6 ± 8.7	22.5 ± 12.6			
**Reversion with WAY-100635**	10.2 ± 4.8	29.9 ± 11.2	22.5 ± 12.1	**Interaction**	$F_{(2, 13)} = 1.79$	n.s.
				**Buspirone**	$F_{(1, 13)} = 1.25$	n.s.
				**Lesion**	$F_{(2, 13)} = 5.51$	$p < 0.05$
**Coefficient of Variation**	**Sham Mean ± SD**	**6-OHDA Mean ± SD**	**6-OHDA/L-DOPA Mean ± SD**	**Two WAY ANOVA**		
**Basal**	29.6 ± 13.3	31.0 ± 13.4	60.3 ± 52.0	**Interaction**	$F_{(8, 52)} = 1.03$	n.s.
**8-OH-DPAT 20**	30.6 ± 22.9	33.6 ± 14.2	67.4 ± 54.9	**Buspirone**	$F_{(4, 52)} = 3.35$	$p < 0.05$
**8-OH-DPAT 40**	38.8 ± 31.5	34.1 ± 14.3	81.7 ± 54.5	**Lesion**	$F_{(2, 13)} = 2.13$	$p < 0.05$
**8-OH-DPAT 80**	48.3 ± 30.6	34.3 ± 15.2	77.8 ± 55.3			

**8-OH-DPAT 160**	29.0 ± 14.5	36.5 ± 16.5	61.8 ± 31.3			
**Reversion with WAY-100635**	61.9 ± 33.1	49.6 ± 18.7	74.5 ± 27.9	**Interaction**	$F_{(2, 13)} = 0.26$	n.s.
				**Buspirone**	$F_{(1, 13)} = 2.63$	n.s.
				**Lesion**	$F_{(2, 13)} = 4.05$	$p < 0.05$
**% effect on ECoG**	**Sham Mean ± SD**	**6-OHDA Mean ± SD**	**6-OHDA/L-DOPA Mean ± SD**	**Two WAY ANOVA**		
**Basal**	100	100	100	**Interaction**	$F_{(8, 52)} = 0.53$	n.s.
**8-OH-DPAT 20**	68.3 ± 33.1	109.7 ± 56.0	83.3 ± 15.1	**Buspirone**	$F_{(4, 52)} = 2.01$	n.s.
**8-OH-DPAT 40**	75.3 ± 30.3	85.12 ± 60.1	64.7 ± 17.4	**Lesion**	$F_{(2, 13)} = 1.77$	n.s.
**8-OH-DPAT 80**	87.4 ± 24.4	89.81 ± 29.6	66.9 ± 16.2			
**8-OH-DPAT 160**	103.5 ± 46.1	113.1 ± 33.1	85.0 ± 53.3			
**% effect on LFP**	**Sham Mean ± SD**	**6-OHDA Mean ± SD**	**6-OHDA/L-DOPA Mean ± SD**	**Two WAY ANOVA**		
**Basal**	100	100	100	**Interaction**	$F_{(8, 52)} = 2.99$	$p < 0.05$
**8-OH-DPAT 20**	117.6 ± 52.2	122.6 ± 18.6	66.2 ± 28.7	**Buspirone**	$F_{(4, 52)} = 2.16$	n.s.
**8-OH-DPAT 40**	91.7 ± 32.6	107.9 ± 37.2	44.0 ± 8.6	**Lesion**	$F_{(2, 13)} = 8.22$	$p < 0.05$
**8-OH-DPAT 80**	119.9 ± 45.6	109.8 ± 38.8	55.0 ± 19.9			

**8-OH-DPAT 160**	85.2 ± 26.4	125.2 ± 49.7	59.2 ± 10.3			
**% effect on coherence EcoG/EP spikes**	**Sham**<br>**Mean ± SD**	**6-OHDA**<br>**Mean ± SD**	**6-OHDA/L-DOPA**<br>**Mean ± SD**	**Two WAY ANOVA**		
**Basal**	100	100	100	**Interaction**	$F_{(8, 52)} = 1.51$	n.s.
**8-OH-DPAT 20**	76.5 ± 13.1	124.9 ± 62.5	115.4 ± 41.5	**Buspirone**	$F_{(4, 52)} = 0.61$	n.s.
**8-OH-DPAT 40**	86.3 ± 34.7	134.0 ± 30.7	113.3 ± 48.9	**Lesion**	$F_{(2, 13)} = 1.63$	n.s.
**8-OH-DPAT 80**	73.5 ± 20.5	144.2 ± 70.6	113.3 ± 40.6			
**8-OH-DPAT 160**	115.9 ± 62.5	130.0 ± 51.2	100.3 ± 47.5			
**% effect on coherence LFP/EP spikes**	**Sham**<br>**Mean ± SD**	**6-OHDA**<br>**Mean ± SD**	**6-OHDA/L-DOPA**<br>**Mean ± SD**	**Two WAY ANOVA**		
**Basal**	100	100	100	**Interaction**	$F_{(8, 52)} = 1.14$	n.s.
**8-OH-DPAT 20**	100.1 ± 20.9	128.3 ± 26.2	71.9 ± 12.2	**Buspirone**	$F_{(4, 52)} = 4.01$	$p < 0.05$
**8-OH-DPAT 40**	125.1 ± 31.7	147.9 ± 7.7	81.7 ± 18.7	**Lesion**	$F_{(2, 13)} = 7.43$	$p < 0.05$
**8-OH-DPAT 80**	153.6 ± 23.6	148.9 ± 14.5	81.75 ± 23.7			
**8-OH-DPAT 160**	167.6 ± 19.9	187.9 ± 23.2	97.9 ± 29.7			

Optostimulation						
	OFF Mean ± SD	ON Mean ± SD	Student's *t* test			
**Protocol 1 - STN**	10.9 ± 3.2	62.2 ± 46.3	t=2.68 df=5		$p < 0.05$	
**Protocol 2 - STN**	12.2 ± 3.8	61.2 ± 35.7	t=3.37 df=5		$p < 0.05$	
**Protocol 3 - STN**	13.1 ± 4.7	39.9 ± 25.6	t=2.59 df=5		$p < 0.05$	
**Protocol 1 - EP**	**OFF Mean ± SD**	**ON Mean ± SD**	**Two WAY ANOVA**			
**Basal**	25.2 ± 13.9	53.6 ± 9.6	**Interaction**	$F_{(1, 7)} = 3.69$		n.s.
**Busp 4 mg/kg**	18.9 ± 12.2	61.0 ± 26.8	**LED**	$F_{(1, 7)} = 25.18$		$p < 0.05$
			**Buspirone**	$F_{(1, 7)} = 0.01$		n.s.
**Protocol 2 - EP**	**OFF Mean ± SD**	**ON Mean ± SD**	**Two WAY ANOVA**			
**Basal**	25.9 ± 11.6	58.4 ± 7.9	**Interaction**	$F_{(1, 7)} = 3.75$		n.s.
**Busp 4 mg/kg**	22.9 ± 11.1	68.8 ± 21.3	**LED**	$F_{(1, 7)} = 32.00$		$p < 0.05$
			**Buspirone**	$F_{(1, 7)} = 0.65$		n.s.
**Protocol 3 - EP**	**OFF Mean ± SD**	**ON Mean ± SD**	**Two WAY ANOVA**			

**Basal**	25.0 ± 10.5	55.8 ± 17.8	**Interaction**	$F_{(1, 7)} = 0.18$	n.s.
**Busp 4 mg/kg**	23.5 ± 11.6	58.4 ± 28.4	**LED**	$F_{(1, 7)} = 18.99$	$p < 0.05$
			**Buspirone**	$F_{(1, 7)} = 0.01$	n.s.
