# Supplementary material for: The effect of 5‐HT1A receptor agonists on the entopeduncular nucleus is modified in 6‐hydroxydopamine‐lesioned rats
Source: Br J Pharmacol. 2021 May 6;178(12):2516–32. doi: 10.1111/bph.15437 (PMC8252460; doi:10.1111/bph.15437)
Supplement: Supplementary file 2 — Table S2. Effect of systemic administration of buspirone on firing properties of entopeduncular neurons [file BPH-178-2516-s004.pdf]

Supplementary Table 2. Effect of systemic administration of buspirone on firing properties of entopeduncular neurons

Sham	Buspirone (mg/kg) i.v.					WAY-101635 (mg/kg) i.v.
	**Basal**	**0.6125**	**1.25**	**2.5**	**5**	**0.5**
**Firing rate (Hz)**	14.6 ± 2.7	7.2 ± 2.3	2.7 ± 1.4*	2.3 ± 1.5*	1.2 ± 0.6*	14.8 ± 1.9
**CV (%)**	51.6 ± 9.9	117.6 ± 45.0*	141.6 ± 47.6*	173.2 ± 58.0*	163.2 ± 46.2*	71.3 ± 9.5
**Neurons exhibiting burst firing pattern (%)**	60	100 &	100 &	100 &	100 &	75 &
**Number of bursts**	19.2 ± 15.2	13.2 ± 6.2	14.2 ± 7.0	18.6 ± 7.6	25.0 ± 11.3	36.2 ± 31.9
**Duration of burst (ms)**	0.8 ± 0.6	2.6 ± 1.2	1.8 ± 0.8	1.8 ±0.7	1.4 ± 0.8	2.7 ± 2.6
**Nº spikes/burst**	10.7 ± 5.3	8.4 ± 3.5	5.4 ± 0.6	5.3 ± 0.7	6.0 ± 1.7	4.4 ± 1.6
**Recurrence of burst (nº burst/min)**	12.7 ± 10.2	8.7 ± 4.2	9.4 ± 4.6	9.4 ± 4.6	16.8 ± 7.6	24.3 ± 22.1
**Intraburst frequency (spike/s)**	31.7 ± 19.9	41.1 ± 19.6	37.0 ± 16.8	36.9 ± 16.8	55.8 ± 31.9	53.6 ± 41.6

Values are expressed as mean ± S.E.M. from sham rats (n = 5). *p < 0.05 vs baseline (RM two-way ANOVA followed by Bonferroni´s post hoc), and &p < 0.05 vs baseline (Fisher´s exact test).

6-OHDA	Buspirone (mg/kg) i.v.					WAY-101635 (mg/kg) i.v.
	**Basal**	**0.6125**	**1.25**	**2.5**	**5**	**0.5**
**Firing rate (Hz)**	20.2 ± 3.0	16.0 ± 1.6	15.4 ± 1.4	15.1 ± 2.0	15.2 ± 3.0	10.8 ± 2.7
**CV (%)**	42.2 ± 4.4	49.0 ± 8.1	48.1 ± 7.8	48.3 ± 6.8	54.5 ± 6.1	43.3 ± 7.0
**Neurons exhibiting burst firing pattern (%)**	66.7	50 $^{\&}$	50 $^{\&}$	66.7	66.7	50 $^{\&}$
**Number of bursts**	14.2 ± 8.7	11.6 ± 5.9	11.6 ± 7.9	10 ± 7.8	12.2 ± 9.7	12.2 ± 10.2
**Duration of burst (ms)**	0.2 ± 0.1	0.4 ± 0.3	0.2 ± 0.1	0.2 ± 0.1	0.2 ± 0.1	0.2 ± 0.1
**Nº spikes/burst**	6.2 ± 3.4	9.5 ± 6.0	5.3 ± 2.3	6.4 ± 2.4	6.1 ± 2.4	5.1 ± 2.1
**Recurrence of burst (nº burst/min)**	8.9 ± 5.3	7.4 ± 5.0	7.4 ± 5.0	6.5 ± 5.0	8.2 ± 6.5	7.9 ± 6.6
**Intraburst frequency (spike/s)**	39.7 ± 10.2	19.4 ± 8.6	19.4 ± 8.6	30.7 ± 9.7	27.8 ± 10.6	22.5 ± 9.9

Values are expressed as mean ± S.E.M. from 6-OHDA-lesioned rats (n = 6). $^{\&}p < 0.05$ vs baseline (Fisher´s exact test).

6-OHDA/L-DOPA	Buspirone (mg/kg) i.v.					WAY-101635 (mg/kg) i.v.
	**Basal**	**0.6125**	**1.25**	**2.5**	**5**	**0.5**
**Firing rate (Hz)**	25.4 ± 3.1	22.1 ± 3.1	18.1± 3.8*	17.8 ± 3.9*	17.1 ± 3.5*	18.8 ± 5.0
**CV (%)**	71.2 ± 17.2	81.3 ± 32.4	111.7 ± 44.6	110.5 ± 40.7	121.0 ± 42.4*	91.4 ± 18.6
**Neurons exhibiting burst firing pattern (%)**	83.3	50 &	66.6 &	83.3	100 &	100 &
**Number of bursts**	55.8 ± 33.1	15.0 ± 9.3	19.3 ± 11.0	32.2 ± 12.3	39.0 ± 12.5	44.3 ± 13.3
**Duration of burst (ms)**	0.3 ± 0.1	0.2 ± 0.1	0.3 ± 0.1	0.3 ± 0.1	0.3 ± 0.1	0.4 ± 0.2
**Nº spikes/burst**	13.2 ± 5.2	9.0 ± 4.2	10.1 ± 3.8	19.2 ± 7.1	10.6 ± 2.1	13.0 ± 2.7
**Recurrence of burst (nº burst/min)**	22.5 ± 11.2	7.5 ± 4.2	32.2 ± 12.3	36.4 ± 8.8	23.4 ± 7.5	27.4 ± 8.2
**Intraburst frequency (spike/s)**	53.9 ± 13.0	26.1 ± 12.0	28.1 ± 9.7	36.4 ± 8.9	44.8 ± 5.7	45.5 ± 8.2

Values are expressed as mean ± S.E.M. from 6-OHDA/L-DOPA rats (n = 6). *p < 0.05 vs baseline (RM two-way ANOVA followed by Bonferroni´s post hoc), &p < 0.05 vs baseline (Fisher´s exact test).
