# Supplementary material for: The effect of 5‐HT1A receptor agonists on the entopeduncular nucleus is modified in 6‐hydroxydopamine‐lesioned rats
Source: Br J Pharmacol. 2021 May 6;178(12):2516–32. doi: 10.1111/bph.15437 (PMC8252460; doi:10.1111/bph.15437)
Supplement: Supplementary file 3 — Table S3. Effect of 8‐OH‐DPAT on firing properties of entopeduncular nucleus (EP) neurons [file BPH-178-2516-s003.pdf]

Supplementary Table 3. Effect of 8-OH-DPAT on firing properties of EP neurons

SHAM	8-OH-DPAT (µg/kg) i.v.					WAY-101635 (mg/kg) i.v.
	Basal	20	40	80	160	0.5
**Firing rate (Hz)**	14.3 ± 2.6	11.4 ± 2.2	8.6 ± 2.4	4.3 ± 1.4*	5.5 ± 1.2	10.2 ± 2.1
**CV (%)**	29.6 ± 5.9	30.6 ± 10.3	38.8 ± 14.1	48.3 ± 13.7	29.0 ± 6.5	49.7 ± 7.6
**Neurons exhibiting burst firing pattern (%)**	40	20 &	0 &	20 &	60 &	60 &
**Number of bursts**	2.5 ± 1.7	1	0	20	4.0 ± 2.5	8.5 ± 7.5
**Duration of burst (ms)**	0.2 ± 0.2	0.8	0	0.1	0.5 ± 0.3	3.3 ± 3.2
**Nº spikes/burst**	3.6 ± 2.8	13	0	1	3.1 ±1.4	6.7 ± 4.1
**Recurrence of burst (nº burst/min)**	1.5 ± 1.0	0.6	0	0.7	2.5 ± 1.5	5.3 ± 4.6
**Intraburst frequency (spike/s)**	43.6 ± 33.8	4.3	0	3.9	17.9 ± 11.2	22.4 ± 10.9

Values are expressed as mean ± S.E.M. from sham rats (n = 5). *p < 0.05 vs baseline (RM two-way ANOVA followed by Bonferroni´s post hoc), &p < 0.05 vs baseline (Fisher´s exact test).

6-OHDA	8-OH-DPAT (µg/kg) i.v.					WAY-101635 (mg/kg) i.v.
	Basal	20	40	80	160	0.5
Firing rate (Hz)	26.4 ± 2.3	29.2 ± 3.6	29.1 ± 4.0	30.4 ± 2.9	30.6 ± 3.9	29.9 ± 5.0
CV (%)	31.1 ± 10.3	33.6 ± 6.3	34.1 ± 6.4	34.3 ± 6.8	36.5 ± 7.4	49.7 ± 7.6
Neurons exhibiting burst firing pattern (%)	60	60	20 [&]	60	60	80 [&]
Number of bursts	6.3 ± 3.3	10.0 ± 9.3	11	8.0 ± 5.5	9.0 ± 4.1	19.5 ± 11.1
Duration of burst (ms)	0.1 ± 0.1	0.3 ± 0.2	0.06	0.1 ± 0.0	0.2 ± 0.1	0.2 ± 0.0
Nº spikes/burst	6.1 ± 3.4	13.1 ± 7.1	3.8	6.7 ± 3.4	10.2 ± 4.6	9.9 ± 3.4
Recurrence of burst (nº burst/min)	4.0 ± 2.2	6.2 ± 5.7	6.6	5.1 ± 3.4	6.1 ± 2.6	12.6 ± 7.4
Intraburst frequency (spike/s)	53.4 ± 21.3	45.2 ± 17.5	16.3	55.9 ± 18.7	57.5 ± 19.2	76.7 ± 10.7

Values are expressed as mean ± S.E.M. from 6-OHDA-lesioned rats (n = 5). [&]$p < 0.051$ vs baseline (Fisher´s exact test).

6-OHDA/L-DOPA	8-OH-DPAT (µg/kg) i.v.					WAY-101635 (mg/kg) i.v.
	Basal	20	40	80	160	0.5
**Firing rate (Hz)**	30.1 ± 5.4	29.5 ± 3.8	26.1 ± 4.3	22.0 ± 5.3*	22.5 ± 5.1	22.5 ± 4.9
**CV (%)**	60.3 ± 21.2	67.4 ± 22.4	81.7 ± 22.7*	77.8 ± 22.6	61.8 ± 12.8	50.1 ± 24.9
**Neurons exhibiting burst firing pattern (%)**	100	50 &	66.67 &	100	100	83.33 &
**Number of bursts**	24.8 ± 15.2	21.2 ± 14.0	25.5 ± 15.3	25.2 ± 10.3	26.5 ± 8.1	15.2 ± 6.8
**Duration of burst (ms)**	0.3 ± 0.1	0.1 ± 0.1	0.1 ± 0.1	0.4 ± 0.1	3.6 ± 3.3	4.4 ± 4.2
**Nº spikes/burst**	15.4 ± 3.9	5.8 ± 3.0	6.8 ± 2.8	12.0 ± 2.3	15.9 ± 5.0	16.3 ± 8.4
**Recurrence of burst (nº burst/min)**	12.5 ± 7.7	13.4 ± 8.7	15.9 ± 9.5	15.6 ± 7.1	17.0 ± 3.8	16.3 ± 8.4
**Intraburst frequency (spike/s)**	67.7 ± 11.5	33.9 ± 17.0	40.9 ± 15.7	55.1 ± 12.1	49.3 ± 14.4	56.7 ± 17.1

Values are expressed as mean ± S.E.M. from 6-OHDA/L-DOPA rats (n = 6). *p < 0.05 vs baseline (RM two-way ANOVA followed by Bonferroni´s post hoc), &p < 0.05 vs baseline (Fisher´s exact test).
