# Supplementary material for: The effect of 5‐HT1A receptor agonists on the entopeduncular nucleus is modified in 6‐hydroxydopamine‐lesioned rats
Source: Br J Pharmacol. 2021 May 6;178(12):2516–32. doi: 10.1111/bph.15437 (PMC8252460; doi:10.1111/bph.15437)
Supplement: Supplementary file 4 — Figure S1. Behavioural tests. (A) Motor asymmetry was evaluated comparing the use of the forelimb contralateral (CL) and ipsilateral (IL) to the lesion in the cylinder test, data are expressed as the mean value of the percentage of ipsilateral or contralateral touches divided into the total number represented as Box and Whiskers representing the median and Min to Max values (*P < .05, two‐tailed paired Student's t‐test). Note that 6‐OHDA‐lesioned animals preferably use the ipsilateral to the lesion. (B) Evolution of dyskinesia scores showing the time course of abnormal involuntary movements (AIMs) scores for axial, limb and orolingual ratings and (C) locomotive score, on the last session after l‐DOPA chronic treatment. All the 6‐OHDA/l‐DOPA animals enrolled in this study developed severe l‐DOPA‐induced dyskinesia (LID). Results are expressed as mean ± S.E.M. [file BPH-178-2516-s002.docx]

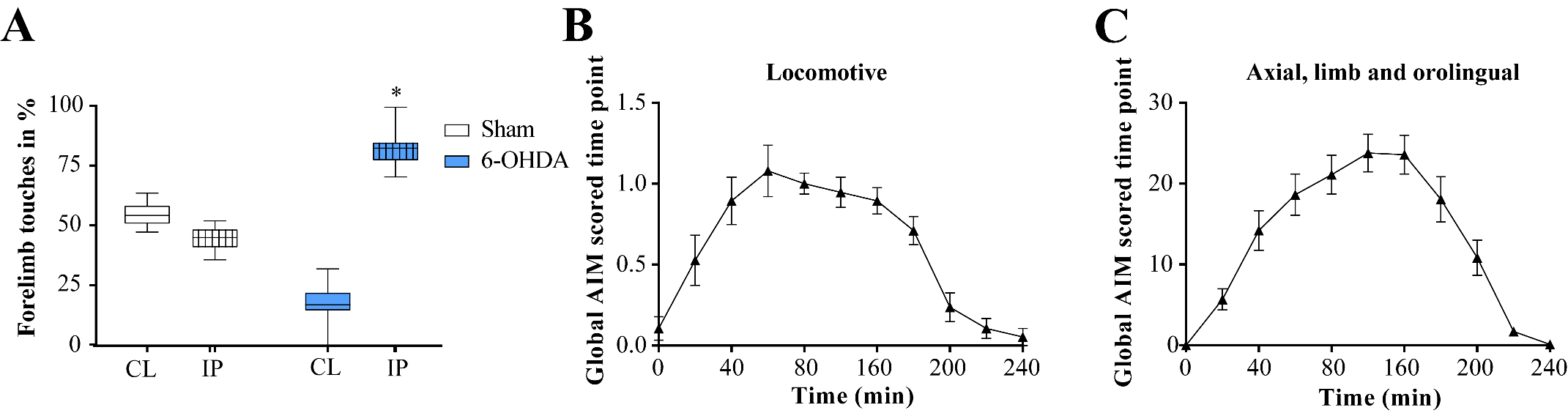


**Figure Supplementary 1. Behavioral tests.** (A) Motor asymmetry was evaluated comparing the use of the forelimb contralateral (CL) and ipsilateral (IL) to the lesion in the cylinder test**,** data are expressed as the mean value of the percentage of ipsilateral or contralateral touches divided into the total number represented as Box and Whiskers representing the median and Min to Max values (*p < 0.05, two-tailed paired Student’s t test). Note that 6-OHDA-lesioned animals preferably use the ipsilateral to the lesion. (B) Evolution of dyskinesia scores showing the time course of abnormal involuntary movements (AIMs) scores for axial, limb and orolingual ratings and (C) locomotive score, on the last session after L-DOPA chronic treatment. All the 6-OHDA/L-DOPA animals enrolled in this study developed severe LID. Results are expressed as mean ± S.E.M.
